# The lower γ region supports forward rotation in the latter half of the 80° substep of F_1_ ‐ATPase

**DOI:** 10.1002/pro.70803

**Published:** 2026-09-23

**Authors:** Tomo Uchiyama, Hiroshi Ueno, Meghna Sobti, Emily J. Furlong, Simon H. J. Brown, Alastair G. Stewart, Hiroyuki Noji

**Affiliations:** ^1^ Department of Applied Chemistry Graduate School of Engineering, The University of Tokyo Bunkyo‐ku Tokyo Japan; ^2^ Heart Rhythms Division The Victor Chang Cardiac Research Institute Sydney New South Wales Australia; ^3^ St Vincent's Clinical School, Faculty of Medicine UNSW Sydney Sydney New South Wales Australia; ^4^ Division of Biomedical Science and Biochemistry, Research School of Biology Australian National University Canberra Australian Capital Territory Australia; ^5^ School of Science, Molecular Horizons, and the Australian Research Council Centre for Cryo‐electron Microscopy of Membrane Proteins University of Wollongong Wollongong New South Wales Australia; ^6^ Research Institute of Planetary Health (RIPH) The University of Tokyo Tokyo Japan

**Keywords:** ATP synthase, chemo‐mechanical coupling, cryo‐electron microscopy, F_1_‐ATPase, rotary molecular motor, single‐molecule analysis

## Abstract

F_1_‐ATPase achieves unidirectional rotation of its γ shaft through coordinated conformational cycling of the α_3_β_3_ ring, yet how shaft–ring interactions contribute to the directionality of rotation remains unclear. Here we analyzed an axle‐less TF_1_, in which the lower γ region is truncated, by combining single‐molecule rotation assays with cryo‐EM structural analysis under catalysis conditions. Under ATP‐saturated conditions, wild‐type TF_1_ exhibited three pauses per turn corresponding to the catalytic dwells, whereas axle‐less TF_1_ exhibited six, revealing an additional intermediate dwell that may be stabilized by γ truncation. This dwell occurred at 40° between the binding and catalytic dwells. High‐speed recordings revealed frequent backsteps during the 40° transition between the intermediate and catalytic dwells. Dwell‐time analysis indicated an approximately zero free‐energy bias between these states, consistent with the absence of a detectable directional bias. Cryo‐EM resolved the corresponding binding, intermediate, and catalytic dwell structures, showing a major β conformational change from 0° to 40°, but minimal β rearrangement between 40° and 80°, suggesting that the rotation is not coupled with a large β conformational transition. Thus, the 0–80° step in axle‐less TF_1_ consists of two regimes: a directional 0–40° motion accompanied by a major β conformational change, followed by a 40–80° interval with little detectable β rearrangement and markedly reduced directional bias. These findings suggest that, in axle‐less TF_1_, the latter half of the 80° substep proceeds through thermal diffusion without directional bias, and that the lower γ region may normally help bias this interval toward forward rotation.

## 1 INTRODUCTION

F_o_F_1_‐ATP synthase (F_o_F_1_) is one of the core enzymes that sustain cellular energy systems. This ubiquitous enzyme is found in the plasma membranes of prokaryotic cells, the thylakoid membranes of chloroplasts, and the inner membranes of mitochondria (Noji et al., 2017; Walker, 2013). F_o_F_1_ synthesizes ATP from ADP and inorganic phosphate (Pi) using the electrochemical potential difference generated across biological membranes, referred to as the proton motive force (*pmf*). The enzyme is composed of two rotary molecular motors, F_o_ and F_1_, that are mechanically coupled (Okuno et al., 2011). F_o_ is a membrane‐embedded motor that generates torque upon proton translocation driven by *pmf*, whereas F_1_ is the membrane‐protruding motor that catalyzes ATP synthesis and hydrolysis (Diez et al., 2004). Through mechanical coupling via a shared central stalk connecting the two rotors and a peripheral stalk linking the stators, F_o_F_1_ interconverts the *pmf* and the free energy of ATP hydrolysis: under high *pmf*, F_o_ forcibly rotates F_1_ in the reverse direction to induce ATP synthesis; when *pmf* diminishes, F_1_ hydrolyzes ATP and drives the reverse rotation of F_o_, pumping protons across the membrane.

When isolated from F_o_, F_1_ functions solely as an ATPase and is therefore referred to as F_1_‐ATPase. The minimal functional composition of F_1_ as a rotary motor is α_3_β_3_γ, in which the central γ subunit rotates against the α_3_β_3_ stator ring (Noji et al., 1997). Three α and three β subunits alternate to form a heterohexameric stator ring penetrated by γ. The α_3_β_3_ ring possesses two distinct types of nucleotide‐binding interfaces: catalytic and non‐catalytic interfaces. At the catalytic interface, most residues essential for ATP hydrolysis reside on β, with a key contribution from an arginine residue on α, termed the arginine finger (Hayashi et al., 2012; Komoriya et al., 2012); accordingly, β is referred to as the catalytic subunit. At the non‐catalytic interface, the nucleotide‐binding site is formed primarily by residues on α, which is therefore termed the non‐catalytic subunit. During catalysis, the three β subunits undergo coordinated conformational transitions associated with their catalytic cycle, collectively generating rotary torque to drive unidirectional rotation of γ (Dai et al., 2017; Masaike et al., 2008; Nishizaka et al., 2004; Sobti et al., 2024; Uchihashi et al., 2011).

The rotation features of F_1_ have been extensively investigated by single‐molecule rotation assays using F_1_ from the thermophilic bacterium *Bacillus* PS3 (TF_1_). In F_1_, γ rotates counterclockwise when viewed from the F_o_ side, with unitary steps of 120° consistent with the pseudo‐three‐fold symmetry of the α_3_β_3_ stator. Each full turn is tightly coupled with three ATP hydrolysis events. The torque generated against viscous friction has been estimated to be approximately 40 pN nm. The high reversibility and efficiency of energy conversion were also experimentally confirmed (Rondelez et al., 2005; Toyabe et al., 2011).

**FIGURE 1:**
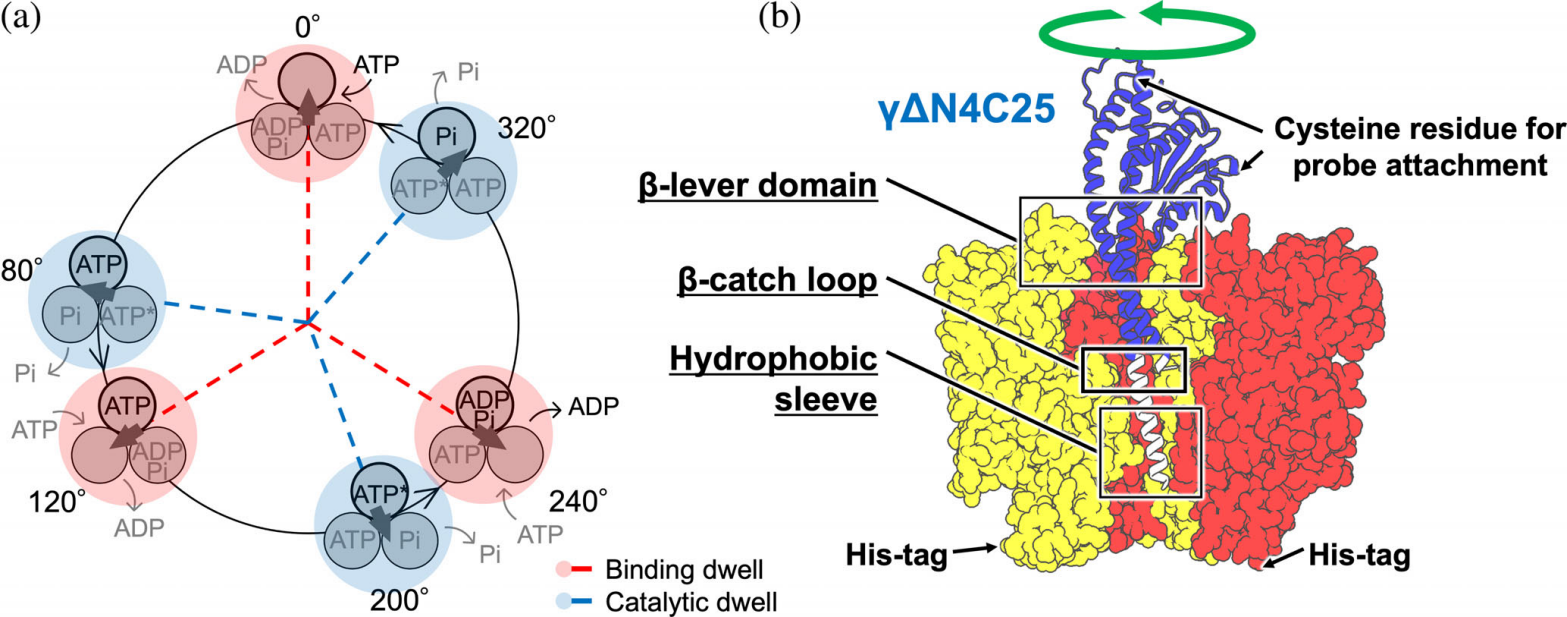
Rotational reaction scheme of wild‐type TF_1_ (WT TF_1_) and the design of axle‐less TF_1_. (a) Rotational reaction scheme of WT TF_1_. The three circles represent the three β subunits in the stator ring, and the nucleotides predicted to occupy the binding sites of each β subunit are shown inside the circles. The thick arrow indicates the rotational angular position of the γ subunit. The 0° position is defined as the γ angle at which a given β subunit binds ATP. An asterisk following “ATP” denotes the catalytically competent state in which the bound ATP undergoes hydrolysis. The angular positions of the binding dwell and catalytic dwell are indicated in red and blue, respectively. (b) Structure of TF_1_ with the deleted N‐terminal 4 and C‐terminal 25 residues of the γ subunit shown in white. The remaining γ region is shown in blue, α in red, and β in yellow. The front αβ pair is removed to show the γ structure. The green arrow indicates the direction of rotation during ATP hydrolysis (counterclockwise as viewed from the membrane side). The black squares indicate the β–γ interaction sites: The hydrophobic sleeve (α280–285, β271–275, and γ271–283), the β‐catch loop (β312–315 and γ262–263), and the β‐lever domain (β382–396, γ15–31, and γ243–253) (Furlong et al., 2025).

The chemomechanical coupling scheme—how rotation and ATP hydrolysis are coupled—has been largely established; each unitary 120° rotation is subdivided into an 80° substep triggered by ATP binding and a subsequent 40° substep associated with ATP cleavage (Shimabukuro et al., 2003; Yasuda et al., 2001). Accordingly, the dwells preceding the 80 and 40° substeps are termed the binding dwell and catalytic dwell, respectively (Figure 1a) (Noji & Ueno, 2022). As a consequence, TF_1_ exhibits six distinct dwells per rotation, during which the three β subunits sequentially undergo ATP hydrolysis, consuming three ATP molecules per turn.

For an individual β subunit, the reaction scheme can be described as follows (Figure 1a). The orientation of the γ subunit when the β subunit awaits ATP binding is defined as 0°, corresponding to one of the binding‐dwell angles. This β subunit hydrolyzes the bound ATP after the γ subunit rotates by approximately 200°, corresponding to a catalytic dwell. ADP release is proposed to occur during the subsequent rotation between 240° and 320° (Adachi et al., 2007; Nishizaka et al., 2004), whereas Pi release is likely to occur at 320° (Adachi et al., 2007; Watanabe et al., 2010).

Previous crystallographic studies predominantly captured F_1_ in the catalytic‐dwell state, leaving the binding‐dwell state structurally unresolved. Cryo‐EM single‐particle analysis (SPA) subsequently captured TF_1_ pausing at the binding dwell, thereby providing the missing structural counterpart to the reaction scheme established by single‐molecule rotation studies (Sobti et al., 2021).

Thus, the chemomechanical coupling reaction scheme has been well established; however, the molecular mechanisms underlying torque generation and transmission within the F_1_ motor remain incompletely resolved. So far, substantial effort has been devoted to elucidating the molecular mechanisms for that purpose. Structural studies have identified three major β–γ interaction sites—the hydrophobic sleeve, the β‐catch loop, and the β‐lever domains—arranged along the γ axle from the N‐terminal side of the α and β subunits (Abrahams et al., 1994; Frasch et al., 2022; Martin et al., 2014). At the hydrophobic sleeve, residues on α and β surround the tip of γ and are thought to function as a bearing, whereas at the β‐lever domains, large conformational changes in the C‐terminal domain of β are proposed to generate torque for the γ rotation.

Early studies focused on specific point interactions between β and γ through site‐directed mutagenesis; however, these studies did not identify residues that were individually crucial for torque generation (Hara et al., 2000; Masaike et al., 2000). Subsequent work therefore employed more extensive mutagenesis approaches, most notably a series of large‐scale truncation analyses of the γ subunit performed by Kinosita's group. These studies showed that even mutants lacking most of the γ axle, retaining only minimal contacts with the β‐lever domain, still exhibited unidirectional rotation, albeit at markedly reduced rotation rate and torque (Furuike et al., 2008; Hossain et al., 2006; Hossain et al., 2008). Importantly, later mutagenesis studies that directly targeted the β‐lever domain itself further demonstrated that disruption of β‐lever–γ interactions does not abolish unidirectional rotation but instead leads to quantitative reductions in rotation rate and torque (Tanigawara et al., 2012). Together, these results indicate that torque generation in F_1_ is mediated by the β–γ interaction as a whole, rather than being transmitted through a few specific interactions. Recent molecular dynamics simulations proposed that the 80° substep rotation is driven by a “distortion–push” mechanism arising from collective deformation of the α_3_β_3_ stator ring. This model is consistent with the idea that flexible and distributed β–γ interactions, rather than a few specific contacts, underlie torque generation in F_1_‐ATPase (Motohashi et al., 2025). However, it has not yet been systematically examined whether these interaction sites contribute uniformly across the entire rotational cycle of F_1_ or whether specific interactions play dominant roles at particular rotational substeps.

Regarding this issue, Furlong et al. (2025) analyzed the structure of the axle‐less TF_1_ mutant that Kinosita's group developed. They resolved the cryo‐EM structure of TF_1_(γΔN4C25); 4 and 25 residues of the N‐ and C‐terminal helices of γ were deleted. This mutant lacks the lower γ shaft region and consequently loses the interactions at the hydrophobic sleeve and β‐catch loop (Figure 1b). The cryo‐EM analysis demonstrated that a canonical binding‐dwell architecture can still be formed despite the loss of these interactions in the lower γ shaft region. However, while this finding established the structural integrity of the binding dwell, it remained unclear how truncation of the γ axle affects subsequent rotational substeps and the directional bias of rotation.

In this study, we examine how truncation of the γ axle influences the rotational behavior of F_1_, with a particular focus on its effects on individual substeps and associated rotary intermediates during chemomechanical coupling. To this end, we exploited the mutant TF_1_(γΔN4C25) with the deletion of the lower γ shaft region that hereafter we refer to as axle‐less TF_1_ for simplicity. By combining single‐molecule rotation assays with cryo‐EM structural analysis under catalytic conditions, we determine how loss of specific β–γ interactions alters rotary intermediates and the associated energy landscape.

## 2 RESULTS

### 2.1 Construct of axle‐less TF_1_ for rotation assays

The expression vector encoding axle‐less TF_1_ was constructed by introducing the γ (ΔN4C25) truncation mutation into the wild‐type TF_1_ (WT TF_1_) with His‐tag at the N‐termini of the α and β subunits and two cysteine residues of the γ subunit for the rotation assay. The expressed truncation mutant F_1_, hereafter referred to as axle‐less TF_1_, was purified according to the previous report (see Methods for details) (Furuike et al., 2008). Gel filtration chromatography of the purified axle‐less TF_1_ protein showed a distinct peak corresponding to the α_3_β_3_γ complex (Figure S1). SDS‐PAGE analysis of the peak fraction confirmed the presence of all component subunits, and the γ subunit was shortened as designed (Figure S1). The intensity of the γ band was lower than that observed for WT TF_1_, as found in the previous report (Furlong et al., 2025), suggesting that a fraction of the complexes lacked the γ subunit. Nevertheless, because sufficient numbers of rotating molecules were obtained in single‐molecule assays, this preparation was judged to be adequate for rotation experiments.

### 2.2 Rotation kinetics and stepping behavior of axle‐less TF_1_

**FIGURE 2:**
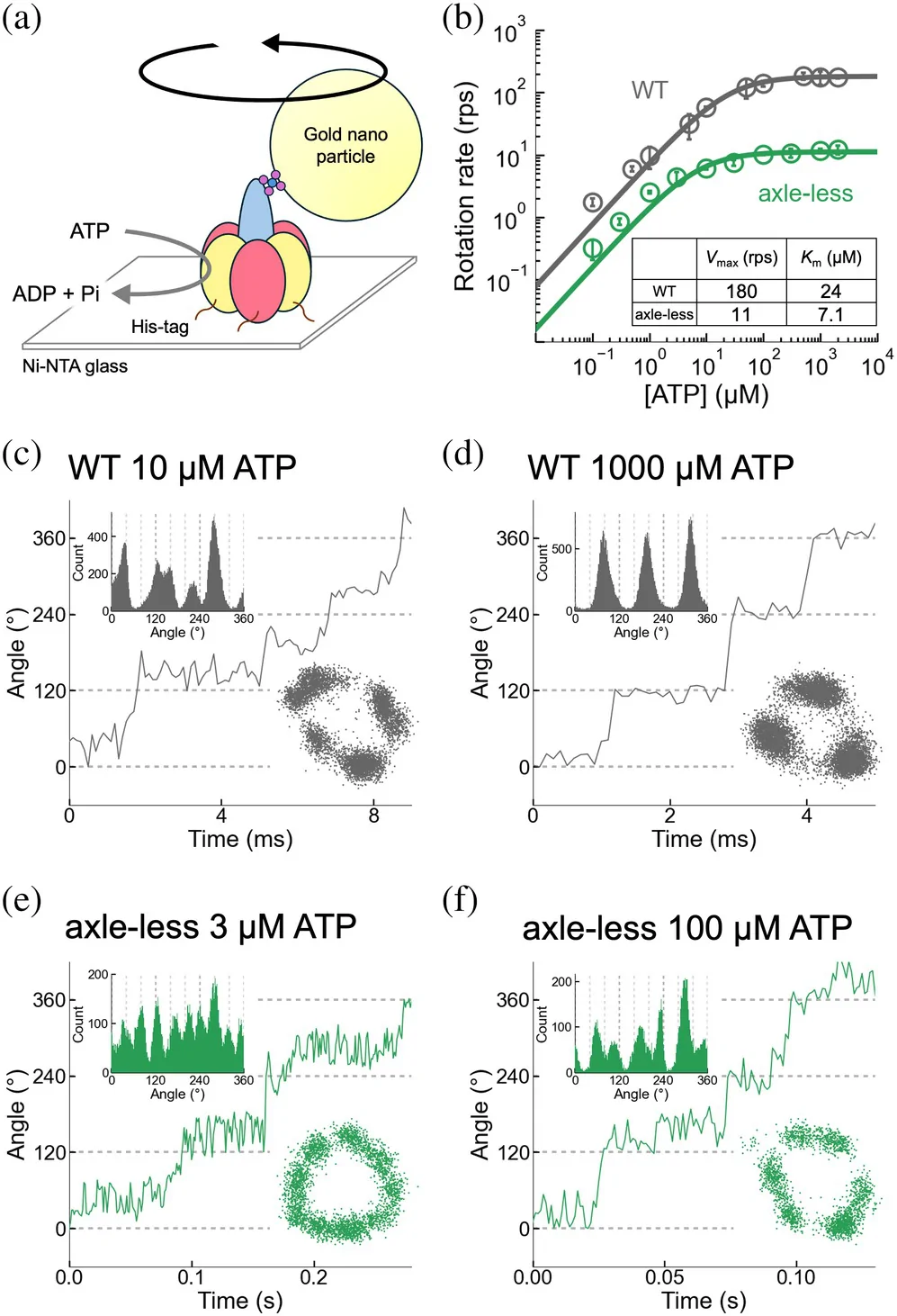
Single‐molecule rotation assays of wild‐type TF_1_ (WT TF_1_) and axle‐less TF_1_. (a) A schematic image of the single‐molecule rotation assay for F_1_. The α_3_β_3_ stator ring was immobilized on a glass surface by His‐tag, and the gold colloid probe was attached to the rotor γ subunit through the biotin‐streptavidin interaction. (b) [ATP] is plotted against the rotation rate for WT TF_1_ (gray) and axle‐less TF_1_ (green). The mean and standard deviation (SD) of each data point are shown as circles and error bars, respectively (*n* = 3–6). Solid lines indicate Michaelis–Menten fits. *V*_max_^WT^: 180 ± 3 rps, *K*_m_^WT^: 24 ± 2 μM. *V*_max_^axle‐less^: 11.2 ± 0.5 rps, *K*_m_^axle‐less^: 7.1 ± 1.8 μM. Values are shown as fitted parameter ± fitting error. (c–f) Representative rotation time courses. The *x*–*y* plots (right) and angular position histograms (left) are shown as insets. (c, f) Rotation of WT TF_1_ recorded at 10,000 fps. (c) 10 μM ATP. (d) 1000 μM ATP. (e, f) Rotation of axle‐less TF_1_ recorded at 1000 fps. (e) 3 μM ATP. (f) 100 μM ATP.

The rotation of axle‐less TF_1_ was observed using 40‐nm gold colloid as the probe under a laser dark‐field microscope (Ueno et al., 2010) (Figure 2a). Rotations were recorded at 10,000 frames per second (fps) for WT TF_1_, and at 500–1000 fps for axle‐less TF_1_ depending on [ATP]. The difference in frame rate reflects the limitations in the on‐board memory of the high‐speed camera; for slowly rotating axle‐less TF_1_, the slower frame rates were chosen to optimally balance sufficient temporal resolution with a sufficiently long recording duration to capture a large number of rotational events within a single acquisition. Figure 2b shows the Michaelis–Menten curves of the rotation rate. The maximum rotation rate (*V*_max_) and Michaelis constant (*K*_m_) were 180 rps and 24 μM for WT TF_1_, and 11 rps and 7.1 μM for axle‐less TF_1_, respectively. The *V*_max_ and *K*_m_ values of axle‐less TF_1_ were approximately one‐tenth and one‐third of those for WT TF_1_, respectively. The ATP‐binding rate constant, *k*_on_, estimated as 3 × *V*_max_/*K*_m_, was determined to be 2.3 × 10^7^ M^−1^ s^−1^ for WT TF_1_ and 4.6 × 10^6^ M^−1^ s^−1^ for axle‐less TF_1_. The *k*_on_ value of axle‐less TF_1_ was approximately one‐fifth of that for WT TF_1_. These results show that axle‐less TF_1_ has slower kinetics, with a reduced ATP‐binding rate and slower catalytic process—ATP cleavage and/or product release.

At low [ATP] far below *K*_m_, axle‐less TF_1_ exhibited three distinctive pauses with 120° intervals, which should correspond to the binding dwells. The histogram of dwell time for ATP binding followed a single exponential decay function (Figure S2). The rate constants determined from the dwell‐time histograms yielded *k*_on_ values of 7.7–7.9 × 10^6^ M^−1^ s^−1^, which agree well with the value estimated from Michaelis–Menten analysis.

Time courses, *x*–*y* plots, and angular position histograms demonstrate stepping rotation of WT TF_1_ and axle‐less TF_1_ (Figure 2c–f). At ATP concentrations above the respective *K*_m_ values, WT TF_1_ showed three pauses with 120° intervals (Figure 2d; Figure S3B), which correspond to the catalytic dwells. In contrast, axle‐less TF_1_ exhibited six characteristic pauses with ~80° and ~40° substeps (Figure 2f; Figure S3D), indicating the presence of additional pauses not observed in WT TF_1_. Around the *K*_m_ range, WT TF_1_ exhibited six pauses with 80° and 40° substeps (Figure 2c; Figure S3A), corresponding to the binding and catalytic dwells. In contrast, axle‐less TF_1_ exhibited nine characteristic pauses with ~40° substeps (Figure 2e; Figure S3C). Importantly, three pauses that became prominent only at low [ATP] emerged at angular positions near the midpoint of the ~80° substeps observed at high [ATP]. Assuming similar angular positions to WT TF_1_, the three pauses following the ~40° substeps observed at high [ATP] are likely to correspond to the catalytic dwells. Taken together, these results suggest that axle‐less TF_1_ exhibits an additional intermediate dwell state between the canonical binding and catalytic dwells that is not observed in WT TF_1_. This point was further confirmed in the following buffer exchange experiments.

Notably, axle‐less TF_1_ frequently made backward steps during rotation (Figure 2e,f; Figure S3C,D). Although the pauses immediately before and after a backward step were estimated to be in the millisecond range, detailed analysis at 1000 fps is not practical. We therefore revisit backward steps later using higher‐frame‐rate recordings.

### 2.3 Identification of the binding dwell angular positions

**FIGURE 3:**
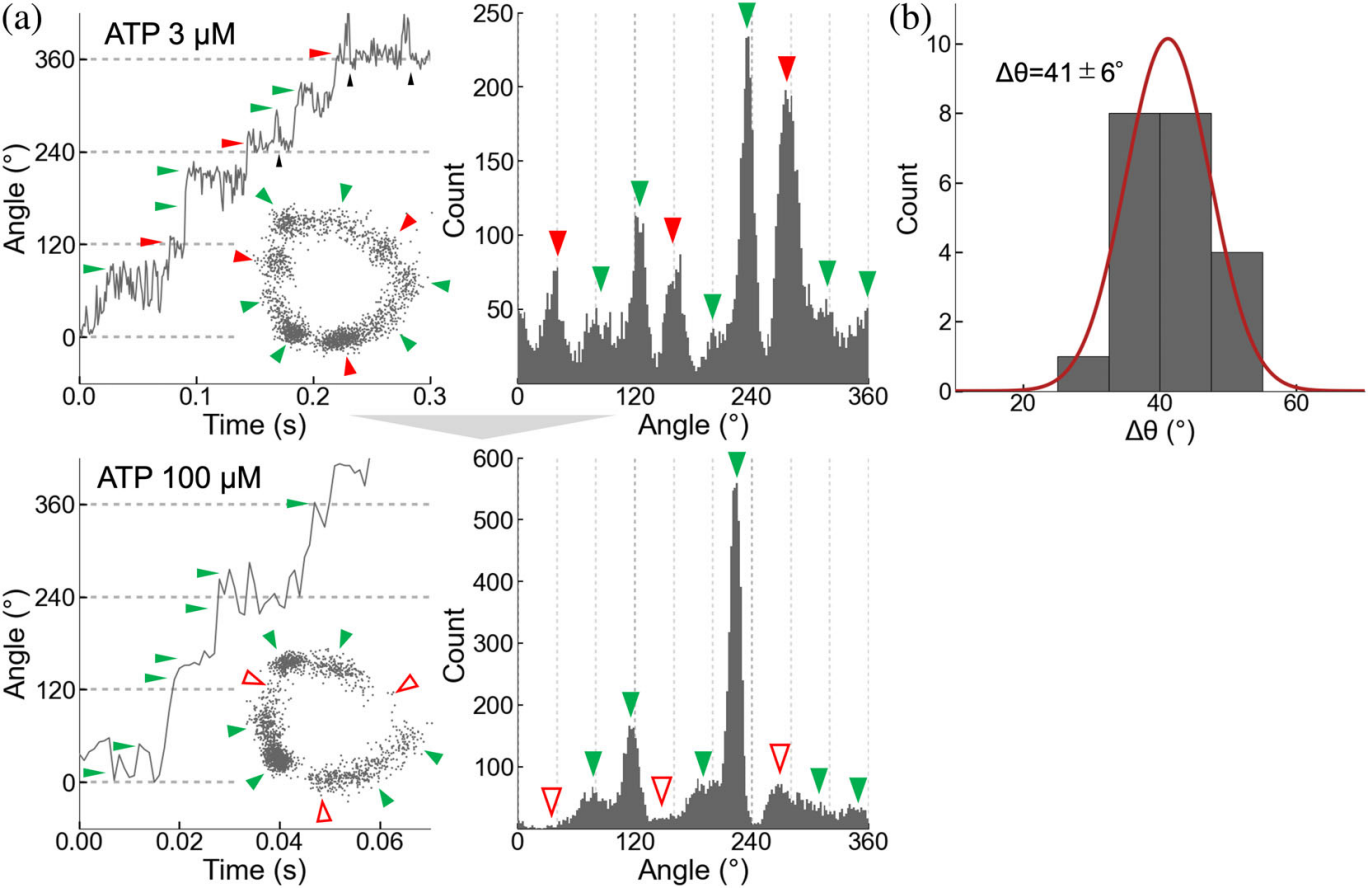
Identification of the binding dwell by buffer exchange experiments. (a) Rotation time courses and angular histograms for the same molecule during a buffer exchange experiment, in which [ATP] was switched between a low concentration around the *K*_m_ range (top) and a high concentration above *K*_m_ (bottom). The *x*–*y* plots are shown as insets. Filled and open red arrowheads indicate observed and expected binding‐dwell positions, respectively. Among the pauses indicated by green arrowheads, three are inferred to correspond to catalytic dwells and the remaining three to an additional dwell state not observed in wild‐type TF_1_ (WT TF_1_). Rare backward‐step‐like events were observed within the 0–40° interval, between the binding dwell and the dwell located 40° ahead. These events are indicated by black arrowheads. Videos were recorded at 1000 fps. (b) Histogram of the angular difference (Δ*θ*) between a binding dwell position and the next dwell position in the direction of rotation. Values are mean ± standard deviation (SD) (*N* = 21, 8 molecules).

To further clarify the angular positions of the binding dwells in axle‐less TF_1_, we performed buffer exchange experiments in which [ATP] was switched between a low concentration around the *K*_m_ range and a high concentration above *K*_m_. By comparing the pause angles under the two conditions, we found that six of the nine pauses observed at low [ATP] overlapped with the pauses at high [ATP] (green arrowheads in Figure 3a; Figure S4), whereas three pauses with 120° intervals almost disappeared at high [ATP] (filled and open red arrowheads in Figure 3a; Figure S4). We therefore assigned the three ATP‐dependent pauses to the binding dwells. The analysis of the angular difference between a binding dwell position and the next dwell position in the rotational direction revealed a histogram centered at 41 ± 6° (Figure 3b). Among the six ATP‐independent pauses, three are expected to correspond to the catalytic dwells and the other three to an additional dwell state not observed in WT TF_1_.

### 2.4 Identification of the catalytic dwell angular positions

ATPγS, a slowly hydrolyzable ATP analog, is known to markedly prolong the catalytic dwell in F_1_‐ATPase (Shimabukuro et al., 2003). We used ATPγS to identify the catalytic dwell of axle‐less TF_1_. Based on the rotational scheme of WT TF_1_, the catalytic dwell is expected to occur ~80° ahead of the binding dwell; however, shortening the γ subunit may shift the catalytic dwell positions. The rotation rate of axle‐less TF_1_ was measured over a range of [ATPγS] values and fitted with a Michaelis–Menten curve (Figure S5). The *V*_max_ and *K*_m_ were determined as 2.8 rps and 1.6 μM, respectively. Although *V*_max_ was reduced to approximately one‐quarter of that for ATP‐driven rotation, this decrease was much smaller than that reported for WT TF_1_, suggesting that the overall turnover of axle‐less TF_1_ is not limited solely by ATPγS‐sensitive reactions.

**FIGURE 4:**
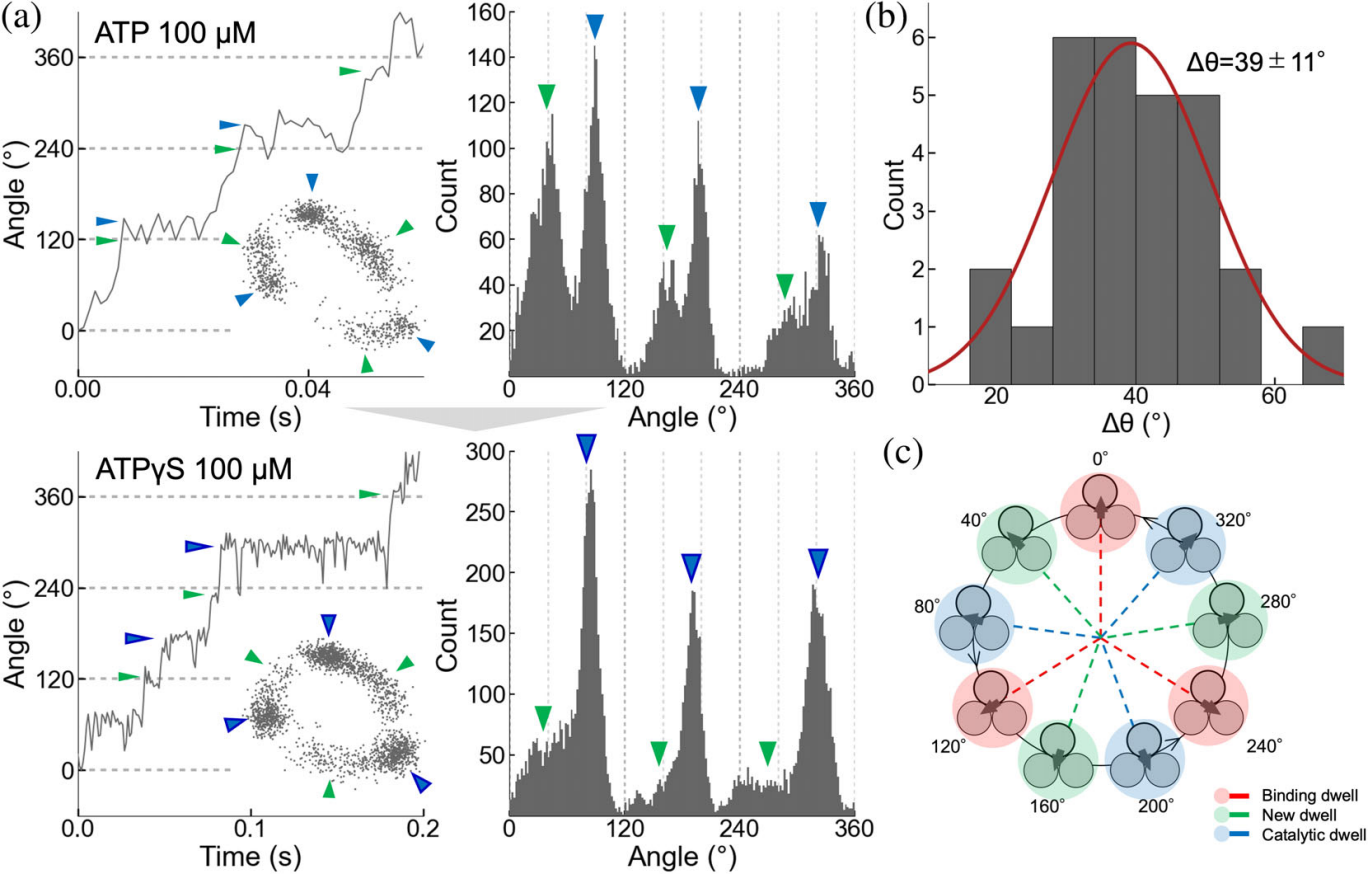
Identification of the catalytic dwell from buffer exchange experiments. (a) Rotation time courses and angular histograms for the same molecule during a buffer exchange experiment, in which the substrate was switched between 100 μM ATP (top) and 100 μM ATPγS (bottom). The *x*–*y* plots are shown as insets. Pauses indicated by blue arrowheads were identified as catalytic dwells. Pauses indicated by green arrowheads were termed the “new dwell,” an additional dwell observed in axle‐less TF_1_. Videos were recorded at 1000 fps. (b) Histogram of the angular difference (Δ*θ*) between the catalytic dwell and new dwell positions. Values are mean ± standard deviation (SD) (*N* = 28, 10 molecules). (c) Rotational reaction scheme of axle‐less TF_1_. The three circles represent the three β subunits in the stator ring. The thick arrow indicates the rotational angular position of the γ subunit. The angular positions of the binding dwell, new dwell, and catalytic dwell are indicated in red, green, and blue, respectively.

We then performed buffer‐exchange experiments to switch the substrate from ATP to ATPγS. Both substrates were used at concentrations above *K*_m_, where axle‐less TF_1_ exhibited ~80° and ~40° substeps (Figure 4a; Figure S6). In ATPγS buffer, axle‐less TF_1_ selectively prolonged the dwell following the ~40° substep (blue arrowheads in Figure 4a; Figure S6). Based on these observations, we assigned the pauses after the ~40° substeps as catalytic dwells (blue arrowheads in Figure 4a; Figure S6) and the three pauses preceding the ~40° substeps, that is, after the ~80° substeps, as an additional dwell state observed in axle‐less TF_1_ (green arrowheads in Figure 4a; Figure S6), which we termed the “new dwell.” The angular difference between the new dwell and the catalytic dwell was centered at 39° (Figure 4b). Together with the abovementioned finding that the new dwell is located ~40° ahead of the binding dwell, these results revealed that axle‐less TF_1_ exhibits binding and catalytic dwell states at the same angular positions as WT TF_1_, with the new dwell located midway between them; at +40° from the binding‐dwell angle and −40° from the catalytic dwell angle (Figure 4c).

### 2.5 Backward step analysis

**FIGURE 5:**
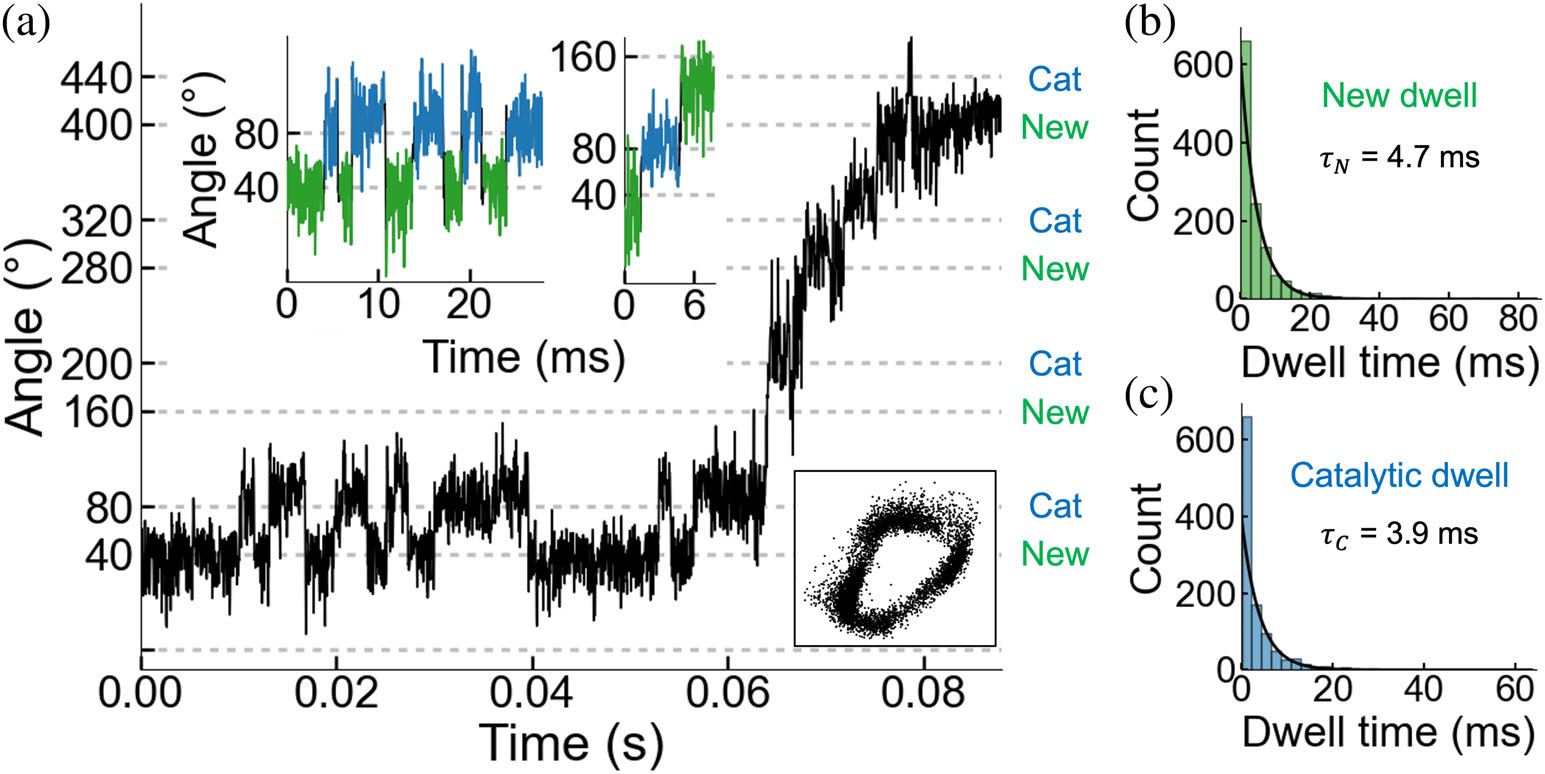
Backward steps between the new dwell and the catalytic dwell at high [ATP]. (a) Representative time course showing a typical backward step during rotation of axle‐less TF_1_ at an ATP concentration above *K*_m_. In the expanded trace, the new dwell and catalytic dwell are indicated in green and blue, respectively. The *x*–*y* plots and expanded time courses are shown as insets. Videos were recorded at 30,000 fps. (b) Histogram of the dwell time for the new dwell, yielding $\tau_{N}$ = 4.7 ± 0.2 ms (*N* = 1253, 3 molecules). (c) Histogram of the dwell time for the catalytic dwell, yielding $\tau_{C}$ = 3.9 ± 0.2 ms (*N* = 1100, 3 molecules). Values are shown as fitted parameter ± fitting error.

For quantitative analysis of backward steps (Figure 2e,f; Figure S3C,D), the rotation of axle‐less TF_1_ was recorded at 30,000 fps. The ATP concentration was set at 100 μM where axle‐less TF_1_ exhibits the new and the catalytic dwells (Figure 5a). The predominant backward event was a backward step from the catalytic dwell to the new dwell located 40° behind. Other backward steps, such as backward steps from the new dwell to the catalytic dwell located 80° behind, as well as backward steps larger than 120° from any dwell, were rarely observed. Within our temporal resolution, forward steps from the new dwell always proceeded via the catalytic dwell, and no direct transitions to the next new dwell were observed. Hereafter, we describe the rotational transitions using three types: (i) a forward 40° step from the new dwell to the next catalytic dwell, (ii) a forward 80° step from the catalytic dwell to the next new dwell, and (iii) a backward 40° step from the catalytic dwell to the new dwell. The reaction scheme and the corresponding rate constants are defined as follows:

$$\mathrm{New}\;\begin{array}{c} k_{N}\\ ⇄\\ k_{b} \end{array}\;\mathrm{Cat}\;\begin{array}{c} k_{f}\\ ⟶\\ \; \end{array}\;\mathrm{New},$$

The histograms of dwell time for the new dwell and the catalytic dwell were each well fitted by a single exponential decay function, yielding time constants $\tau_{N}$ = 4.7 ms and $\tau_{C}$ = 3.9 ms, respectively (Figure 5b,c; Figure S17). The corresponding exit rates were defined as the inverse of these time constants.

$$k_{N}=\frac{1}{\tau_{N}},$$

$$k_{C}=\frac{1}{\tau_{C}},$$

From the catalytic dwell, we counted 80°‐forward steps to the next new dwell ($n_{f}$ = 154) and 40°‐backward steps to the new dwell ($n_{b}$ = 717), giving the forward‐branching probability from the catalytic dwell.

$$P_{f}=\frac{n_{f}}{n_{f}+n_{b}},$$

Based on these values, the backward rate constant from the catalytic dwell was defined as follows:

$$k_{b}=\left(1-P_{f}\right)k_{C},$$

The free‐energy bias between the new dwell and the catalytic dwell along the 40° transition was then estimated from these rate constants.

$$∆G=-k_{B}T\ln\left(\frac{k_{N}}{k_{b}}\right)=-0.01\;k_{B}T,$$

Thus, the new dwell and catalytic dwell states are almost isoenergetic, indicating that the 40° interval between these states is characterized by thermal diffusion without directional bias.

### 2.6 Structural analysis

The structure of axle‐less TF_1_ in the presence of ATP was determined by cryo‐EM, following a previous study (Sobti et al., 2021). Axle‐less TF_1_ has previously been analyzed by cryo‐EM in the presence of AMPPNP as a substrate analogue, and a single state corresponding to the binding dwell was resolved (Furlong et al., 2025). Considering that AMPPNP is a non‐hydrolyzable ATP analog, the obtained conformation may not necessarily represent active rotational intermediates under ATP turnover conditions. To capture conformations that more closely reflect rotary intermediates during ATP‐driven turnover, we re‐examined the structure of axle‐less TF_1_ under ATP substrate conditions.

**FIGURE 6:**
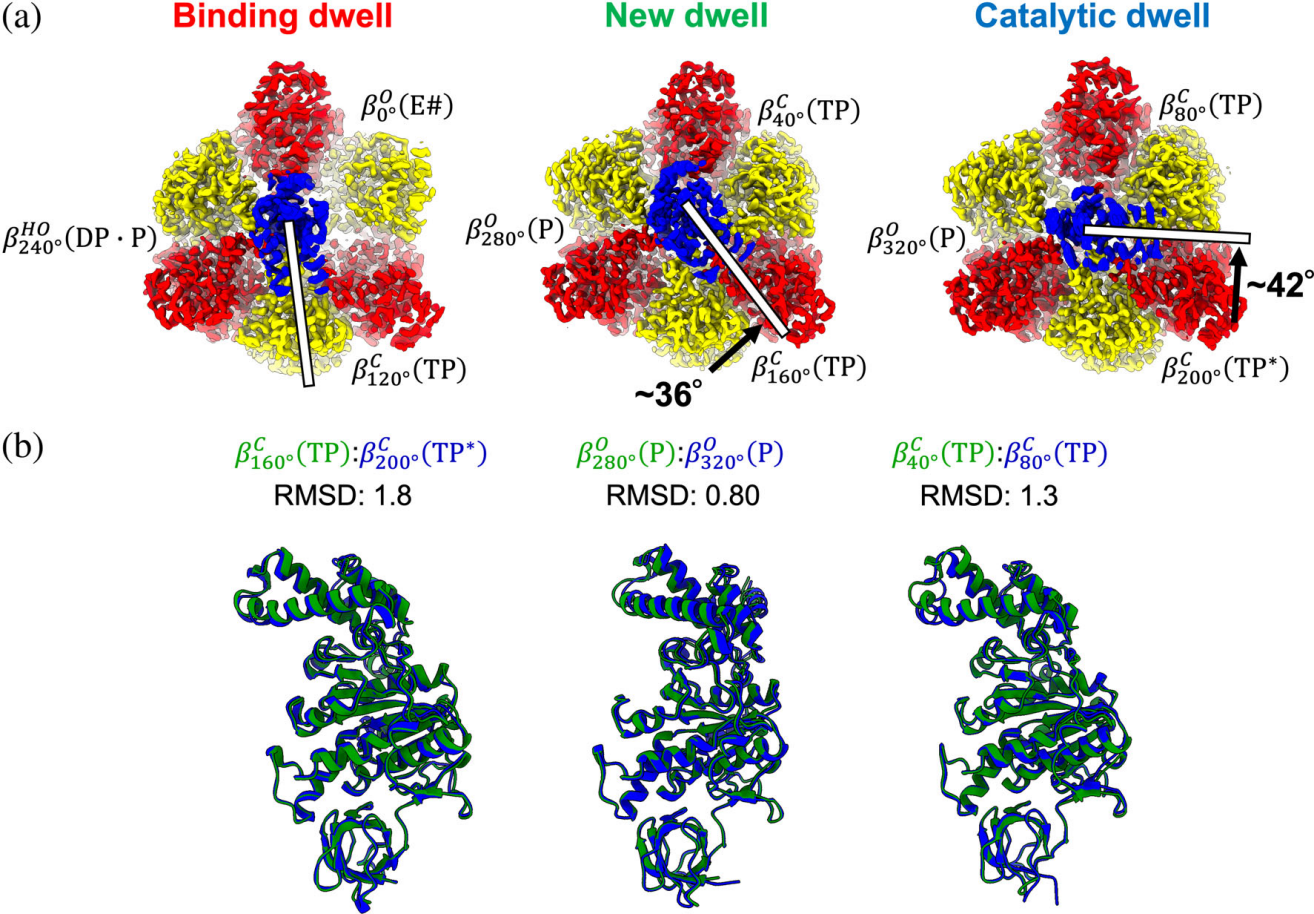
Structure of axle‐less TF_1_. (a) Structures of the three rotational dwells of axle‐less TF_1_. From left to right: Binding dwell, new dwell, and catalytic dwell. Top views are shown, and subunits are colored as in Figure 1b. These structures indicate that the γ subunit rotates counterclockwise among the three dwells (highlighted by a white bar and black arrows). The β subunits are labeled as $\beta_{r{}^{\circ}}^{c}\left(n\right)$, where *c* represents the conformational state of the β subunit, *r*° represents the γ rotation angle relative to the ATP‐binding position defined as 0° in Figure 1a and n represents the nucleotide occupancy of the β subunit (Furlong et al., 2025; Sobti et al., 2021; Sobti et al., 2024; Suzuki et al., 2025). The conformational states are abbreviated as C, HO, O, HC, and AC for closed, half‐open, open, half‐closed, and almost‐closed, respectively. The nucleotide states are abbreviated as TP, TP*, DP, P, E and E# for ATP‐bound, catalytically competent ATP‐bound, ADP‐bound, phosphate‐bound, empty, and empty‐like states, respectively. In E#, E indicates that the canonical nucleotide‐binding site is empty, whereas # indicates weak additional density at a noncanonical phosphate‐binding position, tentatively assigned as phosphate (Figure S11). (b) Comparison of β conformations in axle‐less TF_1_. The β subunit structures of axle‐less TF_1_ new dwell were aligned with the corresponding β structures of axle‐less TF_1_ catalytic dwell, with a focus on the N‐terminal ~81 residues that form the β‐barrel. The new and catalytic dwell structures are shown in green and blue, respectively.

Axle‐less TF_1_ samples purified as described previously (Furlong et al., 2025) were applied to EM grids in the presence of ATP at 22°C, vitrified in liquid ethane, imaged at 300 kV, and processed by SPA. Under these conditions, we obtained three cryo‐EM maps (Figure S7). The map resolutions, estimated by the gold‐standard procedure, were 2.6 Å (binding dwell), 2.6 Å (catalytic dwell), and 2.5 Å (later confirmed as new dwell) (Figure S8). However, these maps were highly anisotropic, with 3D estimates suggesting resolutions around the 3 Å range. Overall, the maps allowed us to define the positions and conformations of each subunit (Figure 6a) and infer the likely nucleotide occupancy (Figure S11). Given the anisotropic nature of these maps, only the cryo‐EM maps were submitted to the EMDB and the models used to interpret the maps are included with this manuscript as Supporting Information.

By comparison with previously reported cryo‐EM structures of TF_1_ (PDB IDs: 7L1Q for the binding dwell and 7L1R for the catalytic dwell) (Sobti et al., 2021), we assigned two of the three maps to the binding dwell and catalytic dwell states, respectively. The remaining map exhibited a new structure in which the γ subunit is oriented at an angle intermediate between the binding and catalytic dwell states; at +36° from the binding dwell structure, and the angular difference between the new dwell and catalytic dwell states was 42°, also consistent with the 39° difference determined by the rotation assays (Figure 6a). Thus, the angular position of the γ subunit in the new structure was well consistent with that of the new dwell. The new structure was interpreted as the new dwell state identified by the single‐molecule rotation assays.

Structural comparison of the β subunits between axle‐less TF_1_ and WT TF_1_ revealed high similarity (Figure S9). In addition, the β conformations in the binding dwell state closely matched those in the previously reported axle‐less TF_1_ binding dwell structure (Furlong et al., 2025) (Figure S10); $\beta_{0{}^{\circ}}$, $\beta_{120{}^{\circ}}$, and $\beta_{240{}^{\circ}}$ take open, closed, and half‐open conformations, respectively. Root‐mean‐square deviation values (Å) represent the structural differences between the aligned models.

Comparison of the new dwell and catalytic dwell structures of axle‐less TF_1_ showed that these two states primarily differ in the rotational angle of the γ subunit, whereas the β subunit conformations remain closely aligned; $\beta_{80{}^{\circ}}$ and $\beta_{200{}^{\circ}}$ take closed conformation, while $\beta_{320{}^{\circ}}$ takes open (Figure 6b). This structural similarity suggests that, unlike the binding‐to‐new transition, the new‐to‐catalytic transition is not accompanied by a large β‐subunit conformational change, although subtle local conformational changes may remain undetected at the present resolution. Together with the backward‐step analysis, these observations indicate that the 40° interval between the new and catalytic dwells is characterized by thermal diffusion without directional bias.

As with nucleotide assignments, the cryo‐EM maps did not contain sufficient detail to assign all residue rotamers de novo. However, residue positions and likely interactions could be inferred from the rotational position of γ and the absence of detectable conformational changes within the subunits relative to previously published structures. Using these models and maps, we compared interactions between the α_3_β_3_ ring and γ in the new dwell and the catalytic dwell. This comparison suggested that the new dwell may contain novel interactions that are absent in the catalytic dwell. When α and β residues located within 4.5 Å of γ were identified, $\beta_{280{}^{\circ}}$ E379–γM29, αE391–γQ22, αA394–γK19, αA394–γI23, and αF398–γI23 were detected exclusively in the new dwell (Figure S15). All the α residues refer to the α subunit positioned between $\beta_{280{}^{\circ}}$ and $\beta_{160{}^{\circ}}$. Among these, $\beta_{280{}^{\circ}}$ E379–γM29 and αA394–γK19 are likely to form van der Waals interactions, αE391–γQ22 a hydrogen bond, and αA394–γI23 and αF398–γI23 hydrophobic interactions. Taken together, these findings suggest that these interactions contribute to stabilization of the rotational pause at the new dwell.

Another aspect found in the new structures of axle‐less TF_1_ is the tilt of the γ subunit. Whereas obvious off‐axis tilt was not found in the binding dwell structure, the catalytic dwell structure exhibits a noticeable tilt. The tilt angles between axle‐less TF_1_ and WT TF_1_ were quantified as 1.3 and 7.5° for the binding dwell and catalytic dwell states, respectively. To test whether the tilted γ orientation could accommodate a full‐length shaft, we aligned the experimentally observed WT TF_1_ γ conformations with the γ subunit of axle‐less TF_1_. This alignment produced steric clashes between the lower region of the full‐length γ subunit and the α_3_β_3_ ring, particularly in the catalytic‐dwell structure (Figure S12). These results indicate that the observed WT γ conformations cannot be accommodated in the tilted axle‐less TF_1_ geometry without additional conformational rearrangement.

### 2.7 Rotational reaction scheme of axle‐less TF_1_

**FIGURE 7:**
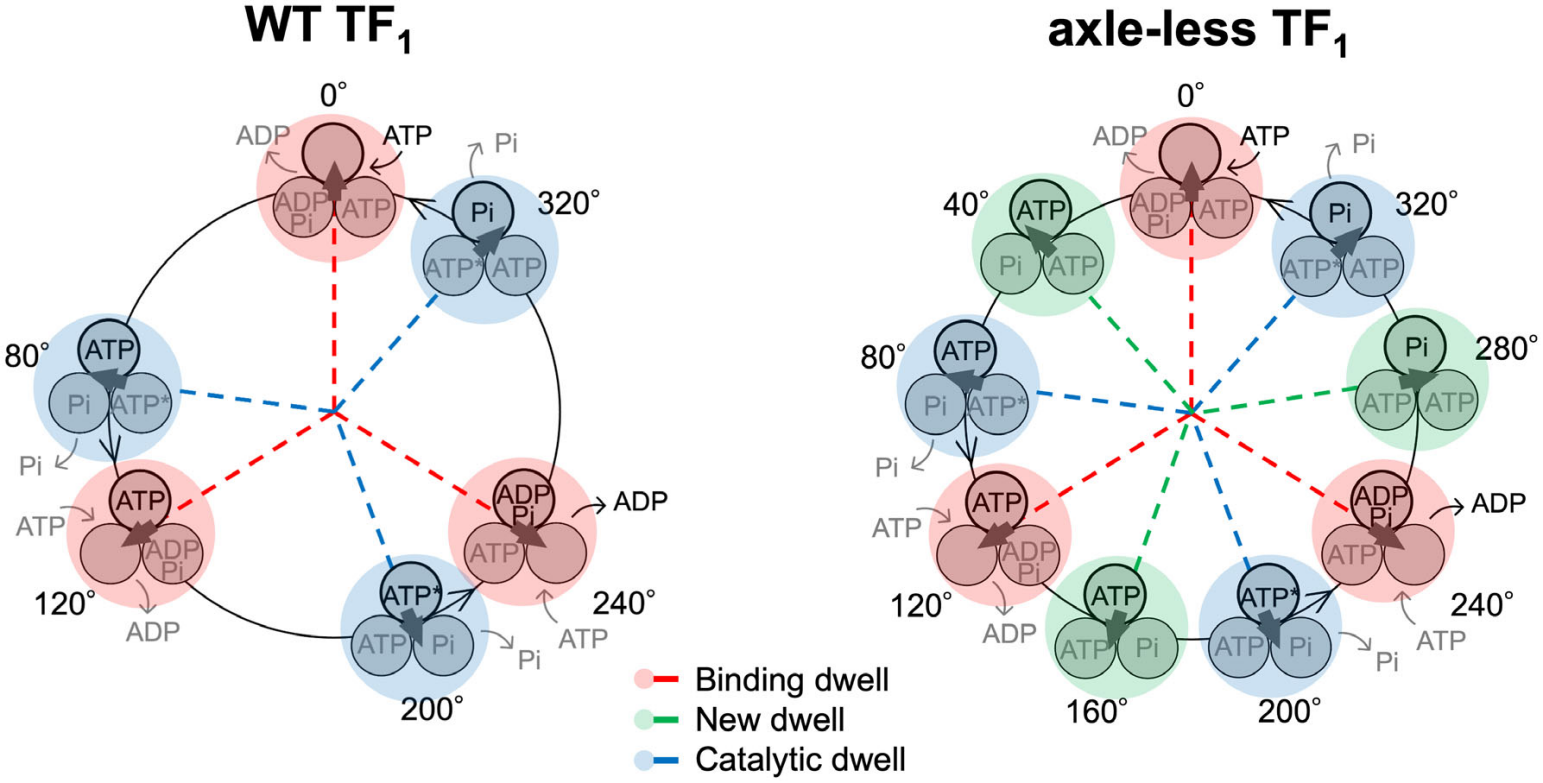
Rotational reaction scheme of wild‐type TF_1_ (WT TF_1_) and axle‐less TF_1_. The left panel shows WT TF_1_, and the right panel shows axle‐less TF_1_. In axle‐less TF_1_, the binding dwell, new dwell, and catalytic dwell are shown in red, green, and blue, respectively. The binding‐ and catalytic‐dwell positions remain aligned with those of WT TF_1_, whereas the new dwell occurs approximately 40° between them. An asterisk following “ATP” denotes the catalytically competent ATP‐bound state. ADP release is shown at the 0° position for simplicity; however, its exact angular position remains unresolved, and the scheme does not imply that ADP release and ATP binding occur simultaneously. Other symbols are as defined in Figure 1a.

Combining the results from single‐molecule rotation assays and cryo‐EM structural analysis, we propose a rotational reaction scheme of axle‐less TF_1_ (Figure 7; Movie S1). The angular positions of the binding and catalytic dwells matched those of WT TF_1_. In addition, axle‐less TF_1_ exhibited an additional intermediate dwell—termed the “new dwell”—located ~40° between the binding and catalytic dwells. In the new dwell state, the conformations of the catalytic β subunits closely match those in the catalytic dwell states 40° ahead. Moreover, the free‐energy difference between the new dwell and catalytic dwell is less than $1k_{B}T$, suggesting that the structural transition between these intermediates can occur through thermal diffusion without directional bias.

Despite their structural similarity, the new dwell and catalytic dwell differ functionally in the catalytic competence of the ATP‐bound site. Accordingly, in the proposed reaction scheme, the ATP‐bound site at the new dwell is denoted as ATP, whereas that at the catalytic dwell is denoted as ATP*, representing the catalytically competent ATP‐bound state. Although the present cryo‐EM maps cannot directly resolve the structural features that distinguish these two ATP‐bound states, our single‐molecule rotation results provide a functional basis for this assignment. In all resolved forward transitions from the new dwell to the next new dwell, the enzyme exhibited a clearly resolved pause at the intervening catalytic dwell. Moreover, ATPγS selectively prolonged the catalytic dwell but not the new dwell. These observations indicate that the ATP‐bound site has not yet reached the catalytically competent state at the new dwell, supporting its assignment as ATP, whereas the corresponding site at the catalytic dwell is assigned as ATP*.

## 3 DISCUSSION

We investigated the functional consequences of truncating the lower γ shaft region by analyzing axle‐less TF_1_. By combining single‐molecule rotation assays with cryo‐EM structural analysis, we identified an additional intermediate dwell in the mutant, termed the “new dwell,” located approximately midway between the binding dwell and the catalytic dwell. High‐time‐resolution single‐molecule measurements further revealed that the transition between the new dwell and the catalytic dwell is reversible, with frequent backward transitions from the catalytic dwell to the new dwell.

Consistent with these observations, cryo‐EM analysis showed that the new dwell and catalytic dwell have highly similar α_3_β_3_ stator‐ring conformations at the domain level, with two β subunits in the closed conformation and one β subunit in the open conformation (the CCO state). No large conformational difference between the two states was detected at the present resolution, although subtle local conformational changes may remain unresolved. These findings indicate that the axle‐less γ subunit can adopt two distinct stable angular positions within the stator ring in the CCO state. The comparable particle populations of the two states further suggest that they are nearly isoenergetic. In contrast, the binding dwell and new dwell structures exhibited clear differences in β‐subunit conformations. Thus, the 0–80° interval of axle‐less TF_1_ can be divided into two regimes: a directional 0–40° motion accompanied by a major β‐subunit conformational change, followed by a 40–80° interval characterized by thermal diffusion without directional bias. Taken together, these results show that truncation of the lower γ region permits thermally driven interconversion between the new and catalytic dwells and thereby reduces the forward bias in the latter half of the 80° substep. This perturbation therefore provides a useful means of revealing the consequences of weakened coupling between the lower γ region and the α_3_β_3_ ring.

The reduced *V*_max_ of axle‐less TF_1_ is likely caused not only by the rate‐limiting process at the catalytic dwell, as in WT TF_1_, but also by the emergence of the new dwell and reversible transitions between the new and catalytic dwells. In axle‐less TF_1_, pauses corresponding to the catalytic dwell were observed under ATP‐saturating conditions, indicating that the catalytic dwell still contributes to rate limitation. However, the new dwell had a dwell time comparable to that of the catalytic dwell, and frequent back‐and‐forth transitions occurred between these two dwells. These processes increase the effective time required for net 120° forward progression and thus likely contribute substantially to the marked reduction in *V*_max_. Consistent with this interpretation, the cryo‐EM structures showed that the new and catalytic dwells share nearly the same α_3_β_3_ stator‐ring conformation and differ mainly in the angular position of γ, suggesting that γ can fluctuate between these two positions without major rearrangement of the catalytic ring. We did not quantitatively compare individual transition durations, including the 0–40° transition, between WT and axle‐less TF_1_ because the measurements were performed at different frame rates. Therefore, possible differences in these transition durations remain unresolved.

Based on these findings, we propose the reaction scheme for axle‐less TF_1_ (Figure 7). Although the current cryo‐EM density around the catalytic site does not allow definitive assignment of the nucleotide chemical states, our results suggest that ATP cleavage occurs not at the new dwell, but at the catalytic dwell. Under ATPγS conditions, the catalytic pause was markedly prolonged in the single‐molecule rotation assay. In addition, high‐frame‐rate recordings showed that forward rotation proceeded after the pause at the catalytic dwell position. Together, these observations support assignment of ATP cleavage to the 200° position. This interpretation is also consistent with the distinction in the rotational scheme of axle‐less TF_1_ (Figure 7) between the ATP‐bound state at $\beta_{160{}^{\circ}}$ in the new dwell and the catalytically competent ATP‐bound state, denoted as ATP* at $\beta_{200{}^{\circ}}$ in the catalytic dwell. Therefore, despite the overall structural similarity between the new dwell and catalytic dwell states, some differences must remain in the catalytic sites. However, the present density maps do not yet allow reliable evaluation of subtle catalytic features, including the configuration of residues directly involved in hydrolysis, such as the arginine finger (Komoriya et al., 2012), or the distinction between ATP and ATP*. Higher‐resolution and more isotropic structural analysis will be required to detect detailed structural differences between the new dwell and catalytic dwell states. Furthermore, identifying which residues in the deleted lower γ region help tighten the ATP‐binding site will require additional experiments combining targeted mutagenesis of candidate residues with single‐molecule rotation assays and structural analysis.

In a previous structural study of axle‐less TF_1_ (Furlong et al., 2025), only the binding dwell state was resolved, whereas structures corresponding to the new dwell and catalytic dwell were not observed. This discrepancy may be attributable to the use of AMPPNP, a non‐hydrolyzable ATP analog. AMPPNP is commonly used to arrest F_1_ rotation at the catalytic dwell, which is thought to correspond to a pre‐hydrolysis state. However, the previous study reported a binding‐dwell structure together with catalytic sites occupied by nucleotide hydrolysis products. These observations raise the possibility that AMPPNP did not fully inhibit the rotation of axle‐less TF_1_ and that residual ATP contamination instead drove the motor under ATP‐binding‐limited conditions. Consistent with this interpretation, our single‐molecule experiments at ATP concentrations far below *K*_m_ detected only three pauses corresponding to the binding dwell, which may explain why the new dwell and catalytic dwell structures were not captured in the previous cryo‐EM analysis.

**FIGURE 8:**
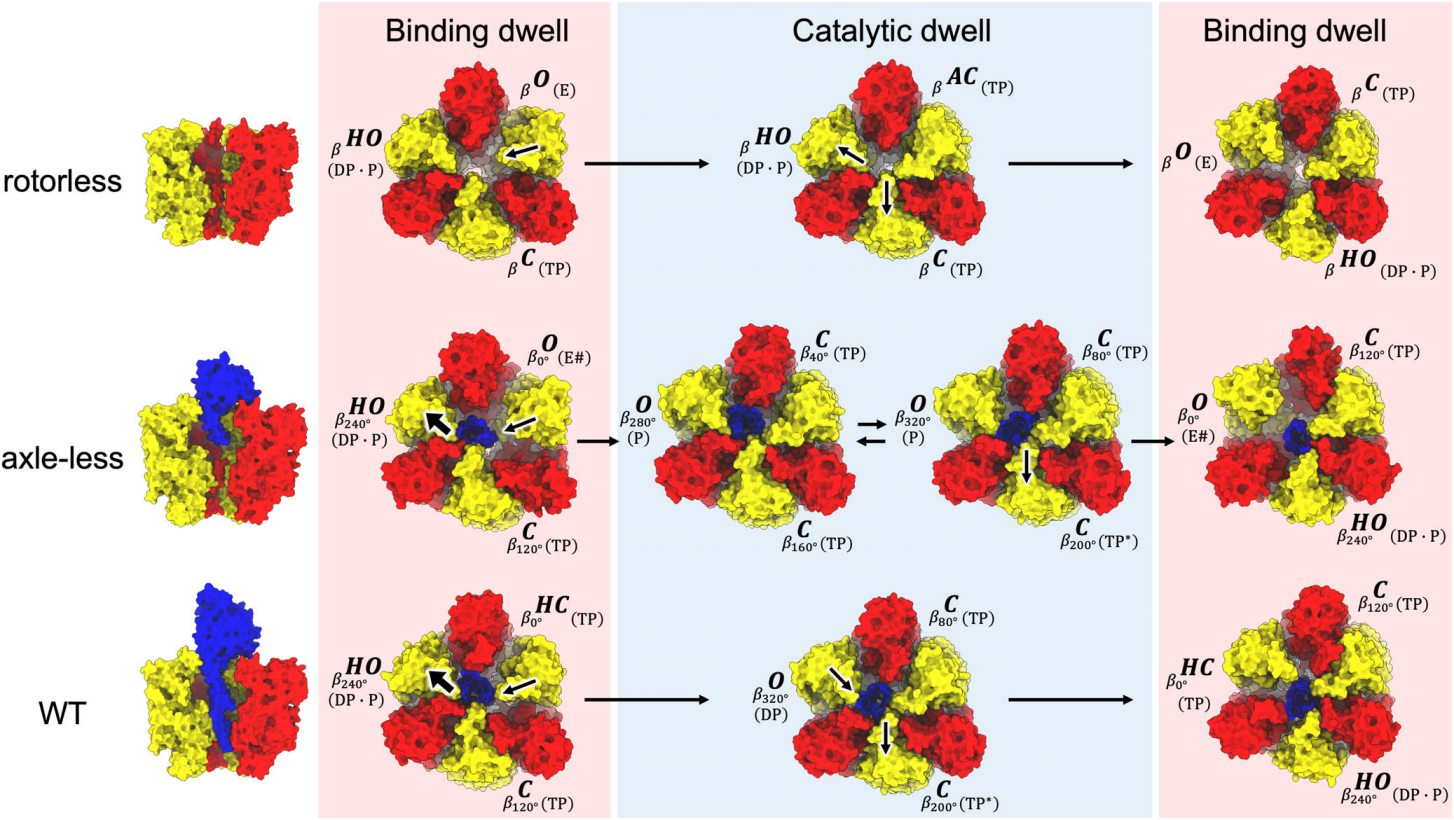
Comparison of the reaction schemes of rotorless, axle‐less, and wild‐type TF_1_ (WT TF_1_). Representative structures are arranged from left to right according to progression of the catalytic cycle. Based on their overall conformations, all structures are broadly classified into binding‐dwell and catalytic‐dwell states, indicated by light red and light blue backgrounds, respectively. For rotorless TF_1_, the states classified here as the binding dwell and catalytic dwell correspond more precisely to the pre‐ATP‐binding and post‐ATP‐binding states, respectively (Sobti et al., 2024). In axle‐less TF_1_, the left structure within the catalytic‐dwell category corresponds to the new dwell. Black arrows on β subunits indicate the principal β‐subunit conformational changes associated with each transition. The thick arrows at $\beta_{240{}^{\circ}}$ in axle‐less and WT TF_1_ highlight the conformational transition of the β subunit from the half‐open state to the open state, proposed to be promoted by the presence of the upper γ region. The rotorless pre‐ and post‐ATP‐binding structures are based on PDB IDs 8SPW and 8SPX, respectively (Sobti et al., 2024), and the WT binding‐ and catalytic‐dwell structures are based on PDB IDs 7L1Q and 7L1R, respectively (Sobti et al., 2021). Subunit colors and β‐subunit notation are as defined in Figures 1b and 6.

Axle‐less TF_1_ can be regarded as an intermediate system between WT TF_1_, which contains the full‐length γ subunit, and rotorless TF_1_, which completely lacks γ (Figure 8). In rotorless TF_1_, sequential ATP hydrolysis is maintained even in the absence of γ, demonstrating that the α_3_β_3_ ring itself possesses intrinsic cooperativity that propagates the catalytic cycle (Dai et al., 2017; Uchihashi et al., 2011). Comparison of the reaction schemes of rotorless, axle‐less, and WT TF_1_ therefore provides a means to identify the net contribution of the remaining upper γ region beyond this intrinsic ring cooperativity. Structural comparison with recently reported rotorless TF_1_ (Sobti et al., 2024) showed that the corresponding β subunit remains in the half‐open state in rotorless TF_1_, whereas it adopts the open state in axle‐less and WT TF_1_ (Figure 8 and Figure S16). This difference suggests that the upper γ region promotes the conformational transition of the β subunit from the half‐open state to the open state. Thus, the presence of the upper γ region alters the conformational states adopted by the β subunits, resulting in a reaction scheme distinct from that of rotorless TF_1_. We interpret these findings to indicate that the upper γ region reinforces the intrinsic cooperativity of the α_3_β_3_ ring and thereby supports more stable and coordinated progression of the catalytic cycle.

The relationship between the new dwell and the rotational pathway of WT TF_1_ requires cautious interpretation. The new dwell may represent a mutant‐specific local minimum stabilized by γ truncation or arise from altered γ–α_3_β_3_ interactions. Nevertheless, previous studies suggest that WT TF_1_ exhibits rapid mechanochemical changes near a similar angular position. The torque profile reported by Frasch and co‐workers exhibited a smooth torque maximum near this angular position but did not reveal a distinct pause or intermediate state corresponding to the new dwell (Sielaff et al., 2016). In contrast, Saita et al. (2015) reported changes in the torque profile at approximately 0°, 40°, and 80° and interpreted the ~40° transition as an intrinsic conformational rearrangement, although no stable pause was observed at this angle. Volkán‐Kacsó et al. (2019) inferred a ~ 10‐μs metastable state approximately 40° after the ATP‐binding dwell from model‐based analysis of high‐speed rotation trajectories and proposed that it may be associated with transient three‐nucleotide occupancy during coupled ATP binding and ADP release. Although the relationship between these previous observations and the new dwell identified here remains unclear, the similar γ angular positions are noteworthy and raise the possibility that γ truncation stabilizes an otherwise short‐lived configuration near this angular position.

One additional structural implication emerged from the present study. Because the new‐dwell structure has not been identified in previous analyses of WT TF_1_ containing the full‐length γ subunit, the new dwell may be stabilized by γ truncation in axle‐less TF_1_. Indeed, when the full‐length γ subunit from WT TF_1_ was inserted into the stator ring of the new‐dwell structure, severe steric clashes with the stator ring were observed, supporting this interpretation (Figure S13). However, this analysis was based on rigid fitting of the experimentally determined WT γ conformations. Because the γ subunit is conformationally flexible, bending or other local structural rearrangements could potentially alleviate these clashes. Moreover, the γ conformations in the binding dwell and catalytic dwell are not identical. We therefore generated an intermediate γ model between these two γ conformations and inserted it into the stator ring of the new‐dwell structure at the +40° angular position. Interestingly, this model structure did not show obvious steric clashes even without introducing the γ tilt (Figure S14). Thus, the clash analysis does not exclude the possibility that a full‐length γ subunit could transiently adopt the +40° angular position through conformational rearrangement. This result suggests that this structural state may, in principle, exist transiently in WT TF_1_.

However, torque was not directly measured in the present study, and although no large β‐subunit conformational change was detected between the new and catalytic dwells, subtle changes may remain unresolved at the present structural resolution. Furthermore, γ rotation without an accompanying conformational transition of the β subunits appears difficult to reconcile with early theoretical studies (Wang & Oster, 1998) and single‐molecule studies (Volkán‐Kacsó et al., 2026; Watanabe et al., 2012), which suggested that γ rotation is tightly coupled with the conformational transitions of the catalytic β subunits associated with changes in the nucleotide affinity. A recent molecular dynamics simulation (Motohashi et al., 2025) also supports the coupling between γ rotation and β conformational transitions. Therefore, future studies will be required to determine whether a corresponding short‐lived state exists in WT TF_1_ and how its structure, nucleotide state, and mechanical role are related to the new dwell observed in axle‐less TF_1_.

## 4 METHODS

### 4.1 Preparation of F_1_

WT TF_1_ for single‐molecule rotation assays was prepared as described previously (Okuno et al., 2008). To attach 40‐nm gold colloids for rotation observation, two cysteine residues were introduced (Watanabe et al., 2023) and the F_1_ complex was biotinylated (Rondelez et al., 2005).

The plasmid encoding axle‐less TF_1_ was constructed using the TF_1_ plasmid pHCXP as the vector and the plasmid encoding γΔN4C25 (pHC95_γΔN4C25) (Furuike et al., 2008) as the insert. After ligation, the recombinant plasmid was transformed into the F_o_F_1_‐deficient *E. coli* strain JM103Δunc. Axle‐less TF_1_ was expressed in *E. coli* and purified and biotinylated using the same procedure as for WT TF_1_. Protein concentration was determined from the absorbance at 280 nm using a molar extinction coefficient of 154,000 M^−1^ cm^−1^.

For cryo‐EM structural analysis, axle‐less TF_1_ protein (γΔN4C25) that was purified for the study by Furlong et al., (2025) was used. The protein was snap frozen and stored at −80°C until utilized in the present study.

### 4.2 Single‐molecule rotation assays

To observe F_1_ rotation, 40‐nm gold colloids (prepared as described in Watanabe et al., (2023) were attached to the biotinylated γ subunit as optical probes.

Flow cells were constructed from two cover glasses (18 × 24 and 24 × 32 mm^2^; Matsunami Glass) using double‐sided tape as a spacer. To capture and immobilize F_1_ by His‐tags introduced into the α_3_β_3_, the bottom glass surface was coated with Ni‐NTA.

The single‐molecule rotation buffer contained 50 mM MOPS‐KOH (pH 7.0), 50 mM KCl, and 2 mM MgCl_2_. When ATP was used as the substrate, an ATP‐regenerating system (100 μg/mL pyruvate kinase and 2.5 mM phosphoenolpyruvate) was included.

For the rotation assay, the flow cell was first filled with rotation buffer containing 5 mg/mL BSA (BSA buffer) and incubated for 5 min. F_1_ in the BSA buffer (WT TF_1_, 200 pM; axle‐less TF_1_, 3 nM) was then introduced and incubated for 5 min, followed by washing with BSA buffer to remove unbound molecules. Next, 40‐nm gold colloids were introduced and incubated for 10 min. Finally, the flow cell was washed with rotation buffer containing substrate to remove unbound particles.

Rotation was observed by dark‐field microscopy (IX‐71, OLYMPUS) with a 60× objective lens and recorded using a high‐speed camera (FASTCAM‐1024PCI, Photron) at 500–30,000 fps at 23°C (Watanabe et al., 2023).

Molecules were selected according to the purpose of each experiment. For rotation‐rate measurements, molecules were included when the center of rotation could be clearly identified from the *x*–*y* trajectory. For buffer‐exchange experiments, stricter criteria were applied: molecules were analyzed only when clear dwell positions were detected in the angular histogram and the rotation showed the expected threefold symmetry. For high‐speed recordings used for step and backstep analysis, molecules were further selected only when individual rotational steps were clearly distinguishable in the time course.

### 4.3 Single‐molecule data analysis

Recorded videos were analyzed using custom software. Pause angles were determined by fitting the angular position histograms with Gaussian functions (Figures 3b and 4b). For backward step analysis, change point analysis was applied to the time traces (Figure 5a) to automate pause detection and averaging, as described previously (Kobayashi et al., 2020; Li et al., 2015). To estimate time constants, the histograms of dwell time were fitted with single exponential decay functions.

### 4.4 Cryo‐EM grid preparation

Briefly, 1 μL of stock solution containing 100 mM ATP and 100 mM MgCl_2_ was added to 4 μL of purified axle‐less TF_1_ and mixed by pipetting, yielding final concentrations of 20 mM ATP and 20 mM MgCl_2_. A 3.5 μL aliquot of the sample was then applied to a glow‐discharged holey gold grid (UltrAuFoil R 1.2/1.3, 200 mesh). The grid was blotted for 4 s at 22°C, using a blot force of 0 and a chamber humidity of 100%, and then plunge‐frozen in liquid ethane using an FEI Vitrobot Mark IV. Approximately 15 s elapsed between ATP addition and sample freezing. This ATP concentration was chosen to maintain ATP‐saturating conditions until vitrification, because axle‐less TF_1_ was estimated to consume ~9 mM ATP during the approximately 15 s interval between ATP addition and freezing.

### 4.5 Data collection

Grids were transferred to a Thermo Fisher Talos Arctica transmission electron microscope (TEM) operating at 200 kV and screened for ice thickness and particle density. Grids were subsequently transferred to a Thermo Fisher Titan Krios TEM G3i operating at 300 kV equipped with a Gatan BioQuantum energy filter (15 eV slit) and K3 Camera at Molecular Horizons, University of Wollongong. Images were recorded automatically using EPU at ×59,000 (displayed magnification of ×105,000 due to the energy filter), resulting in a pixel size of 0.84 Å. A total dose of 74 electrons per Å^2^ was used spread over 80 frames, with a total exposure time of 8.1 s. 13,845 movie micrographs were collected.

### 4.6 Cryo‐EM data analysis

All image processing and refinements were performed in cryoSPARC v4.4.1 (Punjani et al., 2017) using default settings unless otherwise specified. Raw movies were first motion‐corrected, and CTF parameters were estimated using the patch‐based workflow with default parameters (maximum alignment resolution, 5 Å; alignment B‐factor, 500; defocus search range, 1000–40,000 Å). Micrographs with relative ice thickness greater than 1.05 or estimated CTF resolution worse than 4.0 Å were removed to yield a final set of 11,193 movie micrographs. Particles were initially auto‐picked on a subset of the dataset (200 micrographs) using Blob Picker (particle diameter, 100–150 Å) to generate templates for picking. Then, using Template Picker, a total of 7,720,880 particles were picked from the full dataset of 11,193 micrographs. Picked particles were extracted with a box size of 256 pixels, and multiple rounds of 2D classification were performed to exclude “junk” particles derived from aggregates or minor contaminants at the end of each round. After 2D classification, 3,736,633 particles that appeared to represent the complex were retained.

Ab initio and heterogeneous refinement produced three distinct classes: a class corresponding to the binding dwell (608,830 particles), a class corresponding to the catalytic dwell (845,464 particles), and a class corresponding to the new dwell (852,517 particles). Each of the three maps was further subjected to 3D classification using a mask on the rotor γ subunit, and classes with clear γ density were selected. The selected particle numbers were 422,075 for the binding dwell, 415,389 for the catalytic dwell, and 464,363 for the new dwell. Each map was then refined independently by non‐uniform refinement to obtain the final high‐resolution maps. Particle numbers at each step are detailed in the supplementary flowchart (Figure S7), and statistics for all final maps and models are listed in Figure S8 and Table S1. Because some regions exhibited low‐resolution features, DeepEMhancer (Sanchez‐Garcia et al., 2021) was applied to sharpen the maps and improve interpretability for figures showing the entire complex. Non‐uniform refined maps were uploaded to the 3DFSC processing server to obtain additional 3D FSC information.

### 4.7 Model building

Atomic models were built for the intact F_1_ complex and refined using Coot (Emsley et al., 2010), Isolde (Croll, 2018) and PHENIX (Afonine et al., 2018), with the TF_1_ cryo‐EM structures 7L1Q (binding dwell) and 7L1R (catalytic dwell) serving as templates. Due to the anisotropic nature of the maps, the PDB files were not deposited; instead, they are attached as Supporting Information. Figures were prepared using ChimeraX‐1.10.1.

### 4.8 Estimation of the γ subunit rotational angle and tilting

The rotational displacement of the γ subunit between the binding and new dwell states and between the new and catalytic dwell states was measured in ChimeraX‐1.10.1 using the “Measure Rotation” command. The axis of the central γ helix was defined by the Cα atoms of residues 12–42 and 221–257 in chain G. Using this axis definition, the angular differences were 36° (binding to new dwell) and 42° (new to catalytic dwell). Because the γ axis is tilted, these values include an uncertainty of approximately ±5°.

The tilt of the γ subunit was also quantified in ChimeraX‐1.10.1. The γ‐axis was defined with the “define axis” command using the Cα atoms of residues 10–46 and 223–261 in chain G for the binding dwell state, and residues 12–52 and 220–261 in chain G for the catalytic dwell state. For each dwell, the tilt was defined as the angle between the γ‐axis of WT TF_1_ and that of axle‐less TF_1_ and was measured with the “angle” command. Using this procedure, the γ tilt was 1.3° in the binding dwell and 7.5° in the catalytic dwell.

### 4.9 Construction of TF_1_ models with an artificially 40°‐rotated γ subunit

Models were generated in which the γ subunit of TF_1_ was artificially rotated by +40° in the binding dwell structure (PDB ID: 7L1Q) and by −40° in the catalytic dwell structure (PDB ID: 7L1R). All coordinate transformations and structure manipulations were performed using custom Python scripts with MDAnalysis. First, each F_1_ structure was translated so that the center of the three centers of mass (Cα atoms only) of the N‐terminal domains (residues 3–83) of the three β subunits became the origin. Next, the structure was rotated so that the *z* axis became perpendicular to the plane defined by these three centers of mass. The F_1_ complex was then rotated around the *z* axis so that the center of mass of the N‐terminal domain (residues 3–83) of the β subunit (chain D) lay on the *x* axis, thereby aligning the α_3_β_3_ ring in a common reference frame. After ring fitting, the coordinates of the γ subunit (chain G) were rotated about the *z* axis passing through the origin by applying a rotation matrix $R_{z}\left(\theta \right)$ only to γ, while keeping the α and β subunits fixed. The rotation angle was set to θ = +40° for the 7L1Q‐derived model and θ = −40° for the 7L1R‐derived model. The rotated γ coordinates were then merged back with the unmodified ring coordinates to generate TF_1_ models with an artificially rotated γ subunit.

## AUTHOR CONTRIBUTIONS

**Tomo Uchiyama:** Conceptualization; investigation; methodology; data curation; formal analysis; validation; visualization; writing – original draft; writing – review and editing. **Hiroshi Ueno:** Writing – review and editing; methodology; conceptualization; funding acquisition. **Meghna Sobti:** Formal analysis; investigation; resources; visualization; writing – review and editing; data curation; methodology. **Emily J. Furlong:** Resources. **Simon H. J. Brown:** Resources; formal analysis; data curation; investigation. **Alastair G. Stewart:** Methodology; formal analysis; resources; visualization; funding acquisition; writing – review and editing; data curation; investigation. **Hiroyuki Noji:** Writing – review and editing; funding acquisition; methodology; supervision; conceptualization; project administration.

## CONFLICT OF INTEREST STATEMENT

The authors declare no conflicts of interest.

## Supporting information


**Figure S1.** Gel filtration chromatogram and SDS‐PAGE analysis of axle‐less TF_1_.
**Figure S2.** Time‐constant analysis at low [ATP] far below *K*_m_.
**Figure S3.** Single‐molecule rotation assays of WT TF_1_ and axle‐less TF_1_, related to Figure 2.
**Figure S4.** Identification of the binding dwell from buffer exchange experiments, related to Figure 3.
**Figure S5.** [ATPγS] versus rotation rate.
**Figure S6.** Identification of the catalytic dwell from buffer exchange experiments, related to Figure 4.
**Figure S7.** Cryo‐EM data collection and data processing flowchart.
**Figure S8.** 3D FSC Curves and Local Resolution Estimates.
**Figure S9.** Comparison of β conformations in axle‐less TF_1_.
**Figure S10.** Comparison of binding dwell β conformations between the axle‐less TF_1_ structure determined in this study and the previous study.
**Figure S11.** Close‐up views of the nucleotide binding sites.
**Figure S12.** Steric clashes between γ and αβ upon alignment of TF_1_ γ onto axle‐less TF_1_ γ.
**Figure S13.** Steric clash between the TF_1_ catalytic dwell α_3_β_3_ ring and γ subunits at 40° rotational angle.
**Figure S14.** Fitting of full‐length γ in the new‐dwell α_3_β_3_ conformation of axle‐less TF_1_.
**Figure S15.** New interactions between α/β and γ in the new dwell.
**Figure S16.** Comparison of β conformations in axle‐less TF_1_ and rotorless TF_1_.
**Figure S17.** Molecule‐by‐molecule dwell‐time analysis of the new and catalytic dwells.
**Table S1.** Cryo‐EM data collection, refinement and validation statistics.
**Table S2.** Parameters used to estimate the free‐energy bias between the new and catalytic dwells for individual molecules and merged data.

**Data S1.** Binding_dwell_model.pdb.

**Data S2.** New_dwell_model.pdb.

**Data S3.** Catalytic_dwell_model.pdb.

**Movie S1.** Structural animation of axle‐less TF_1_.

## Data Availability

Cryo‐EM maps have been deposited under Electron Microscopy Data Bank (EMDB) codes EMD‐69782, EMD‐69783, and EMD‐69784. Molecular models are included as Supporting Information.
