# Supplementary material for: The lower γ region supports forward rotation in the latter half of the 80° substep of F1 ‐ATPase
Source: Protein Sci. 2026 Sep 23;35(10):e70803. doi: 10.1002/pro.70803 (PMC13599361; doi:10.1002/pro.70803)
Supplement: Supplementary file 1 [file PRO-35-e70803-s003.pdf]

Supporting Information

The Lower γ Region Supports Forward Rotation in the Latter Half of the 80° Substep of F_1-ATPase

Tomo Uchiyama[1], Hiroshi Ueno[1]*, Meghna Sobti[2,3], Emily J. Furlong[2,4], Simon H. J. Brown[5], Alastair G. Stewart[2,3], Hiroyuki Noji[1,6]*

[1]Department of Applied Chemistry, Graduate School of Engineering, University of Tokyo.

[2]Heart Rhythms Division, The Victor Chang Cardiac Research Institute, NSW, Australia.

[3]St Vincent's Clinical School, Faculty of Medicine, UNSW Sydney, NSW, Australia.

[4]Division of Biomedical Science and Biochemistry, Research School of Biology, Australian National University, Canberra, ACT, Australia.

[5]School of Science, Molecular Horizons, and the Australian Research Council Centre for Cryo-electron Microscopy of Membrane Proteins, University of Wollongong, Wollongong, NSW, Australia.

[6]Research Institute of Planetary Health (RIPH), The University of Tokyo.

*Corresponding authors: hueno@g.ecc.u-tokyo.ac.jp and hnoji@g.ecc.u-tokyo.ac.jp

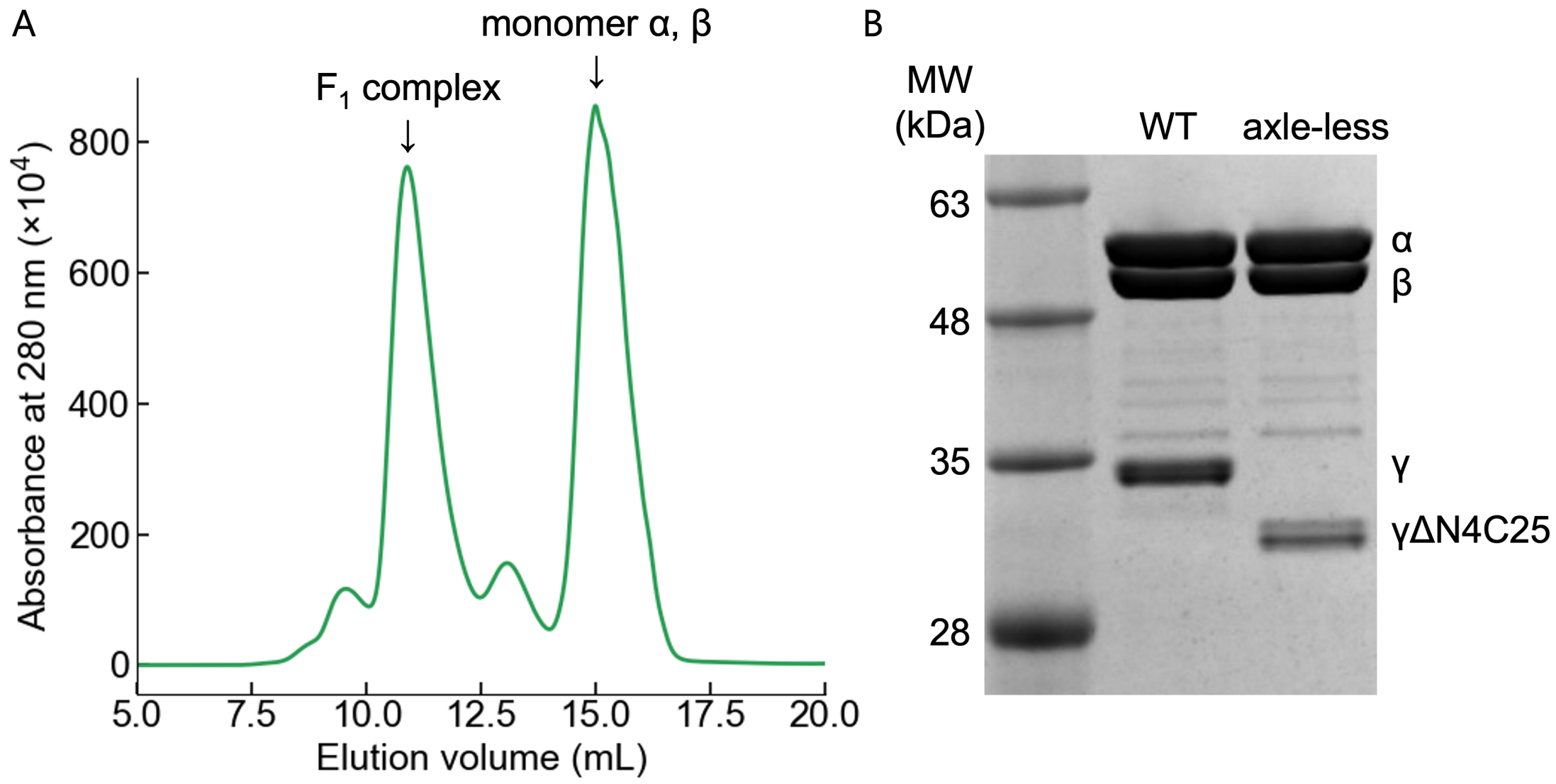

Fig. S1. Gel filtration chromatogram and SDS-PAGE analysis of axle-less TF_1.

(A) Gel filtration chromatogram of axle-less TF_1 after Ni-NTA purification. The peak corresponding to the F_1 complex eluted at nearly the same position as the peak observed during purification of WT TF_1 with a comparable molecular weight. Peaks corresponding to α and β monomers were also detected. (B) SDS-PAGE analysis of axle-less TF_1. From left to right, lanes correspond to the marker, WT TF_1, and axle-less TF_1. WT TF_1 and axle-less TF_1 were loaded at the same concentration. The γ subunit of axle-less TF_1 was detected at a lower apparent molecular weight than that of WT TF_1. The α and β band intensities were comparable between WT TF_1 and axle-less TF_1, whereas the γ band was weaker in axle-less TF_1, consistent with reduced γ content in axle-less TF_1. The γ subunit showed doublet bands due to partial biotinylation, as reported previously[1].

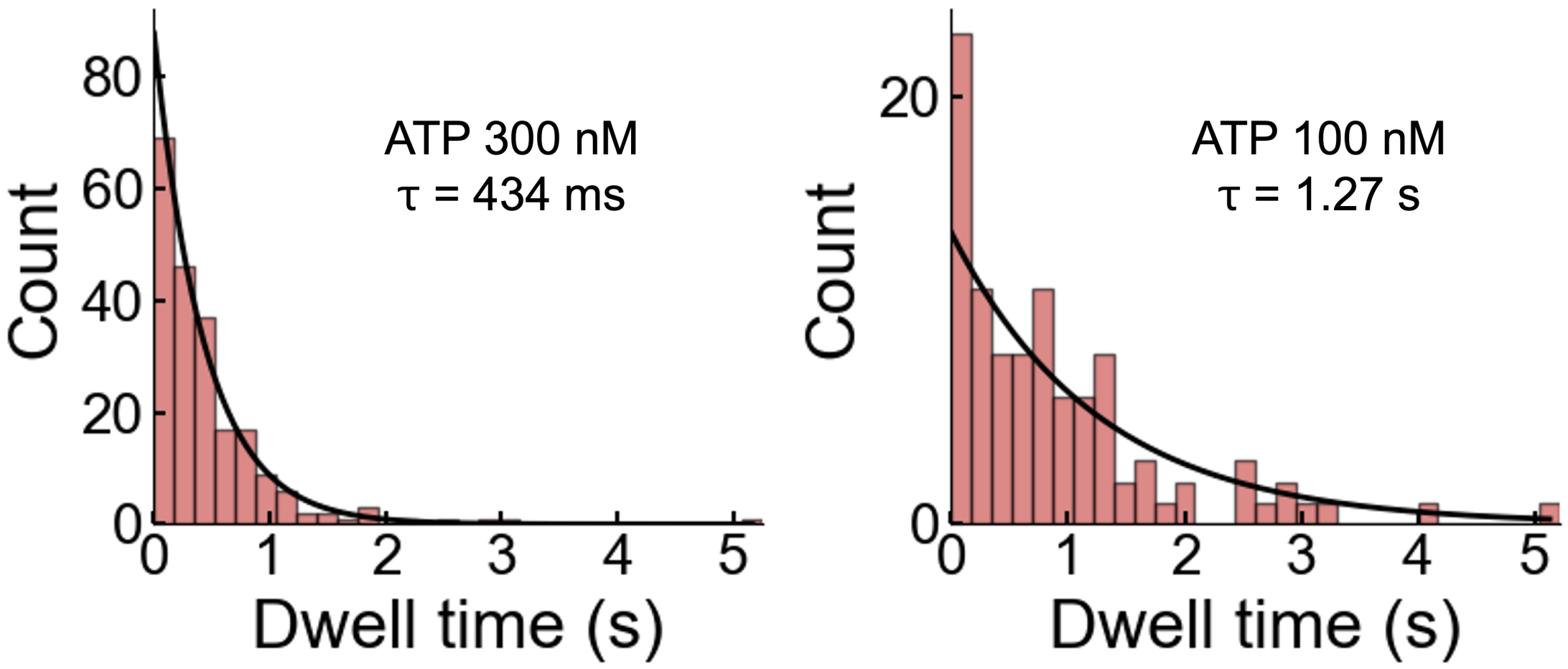

Fig. S2. Time-constant analysis at low [ATP] far below K_{m}.

Histograms of the dwell time at 300 nM ATP (N = 214, 4 molecules) and 100 nM ATP (N = 99, 4 molecules). Single-exponential fitting yielded $\tau = 434 \pm 32$ ms at 300 nM ATP and $\tau = 1.27 \pm 0.21$ s at 100 nM ATP. Values are shown as fitted parameter ± fitting error. Videos were recorded at 500 fps.

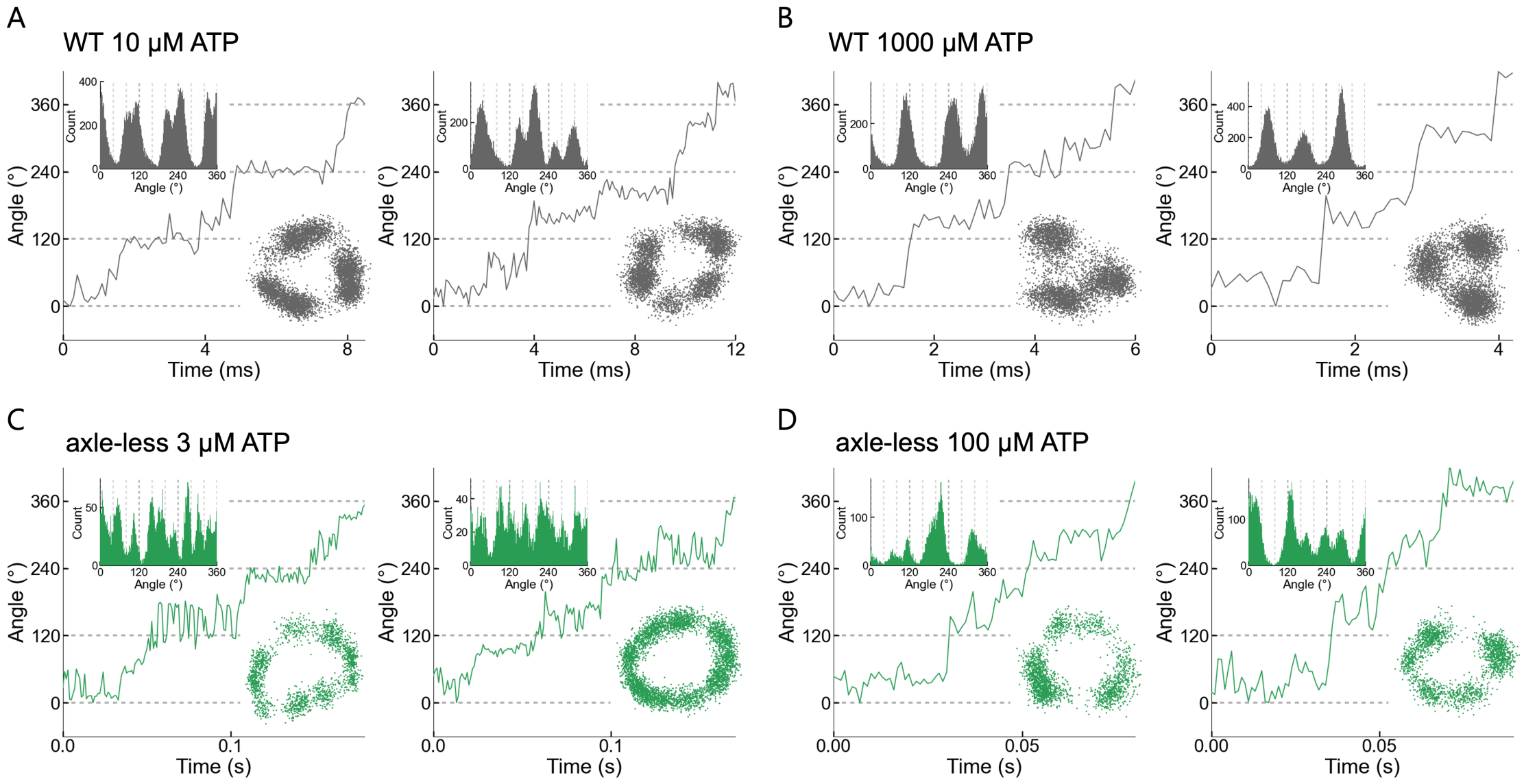

Fig. S3. Single-molecule rotation assays of WT TF_1 and axle-less TF_1, related to Fig. 2.

Representative rotation time courses. The x–y plots (right) and angular position histograms (left) are shown as insets. Two molecules are shown as examples for each condition. (A, B) Rotation of WT TF_1 recorded at 10,000 fps. (A) 10 µM ATP. (B) 1000 µM ATP. (C, D) Rotation of axle-less TF_1 recorded at 1,000 fps. (C) 3 µM ATP. (D) 100 µM ATP.

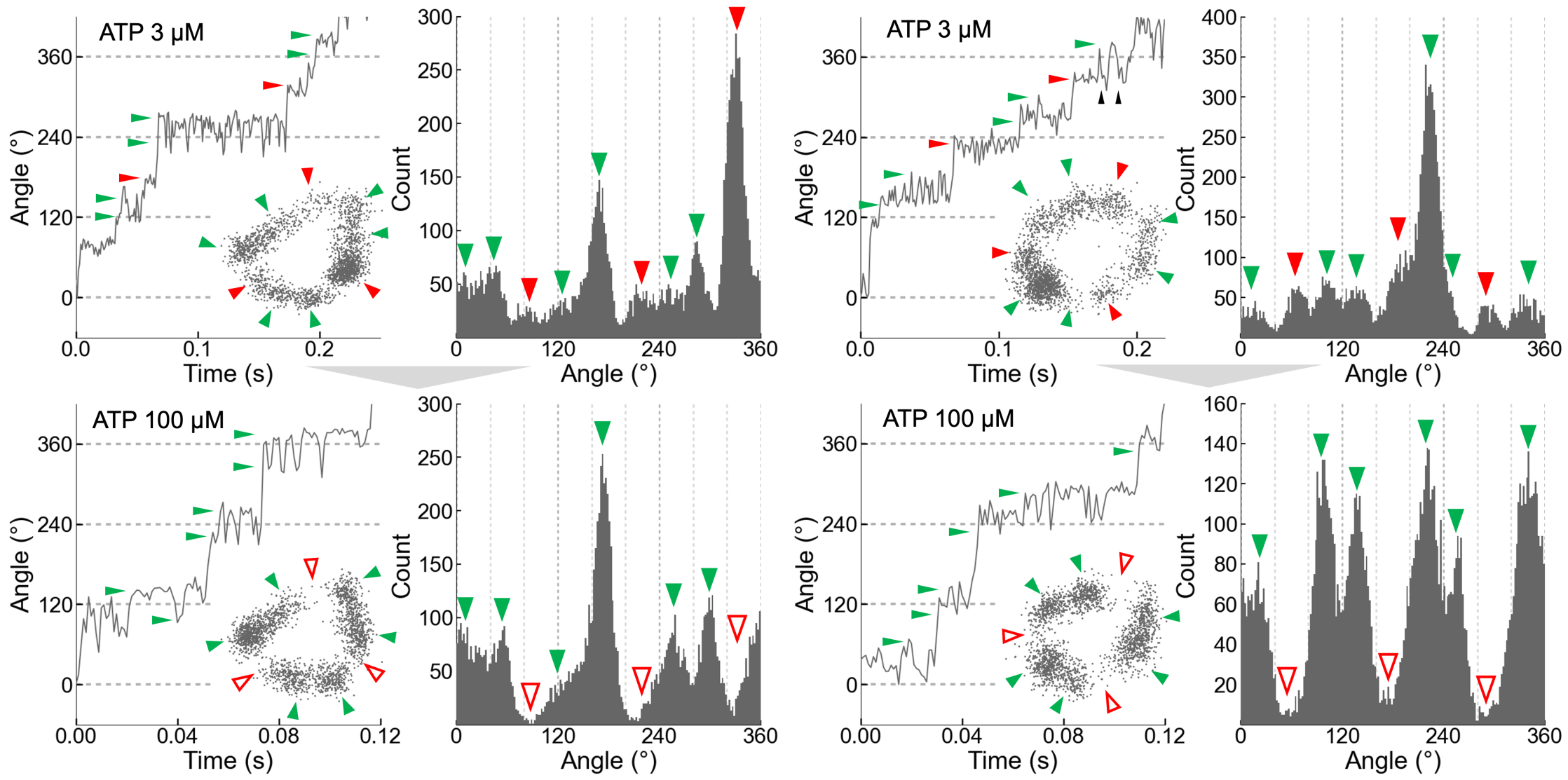

Fig. S4. Identification of the binding dwell from buffer exchange experiments, related to Fig. 3.

Rotation time courses and angular histograms for the same molecule during a buffer exchange experiment, in which [ATP] was switched between a low concentration around the K_m range (top) and a high concentration above K_m (bottom). The x–y plots are shown as insets. Two molecules are shown as examples. Filled and open red arrowheads indicate observed and expected binding-dwell positions, respectively. Among the pauses indicated by green arrowheads, three are inferred to correspond to catalytic dwells and the remaining three to an additional dwell state not observed in WT TF_1. Rare backward-step-like events were observed within the 0–40° interval, between the binding dwell and the dwell located 40° ahead. These events are indicated by black arrowheads. Videos were recorded at 1,000 fps.

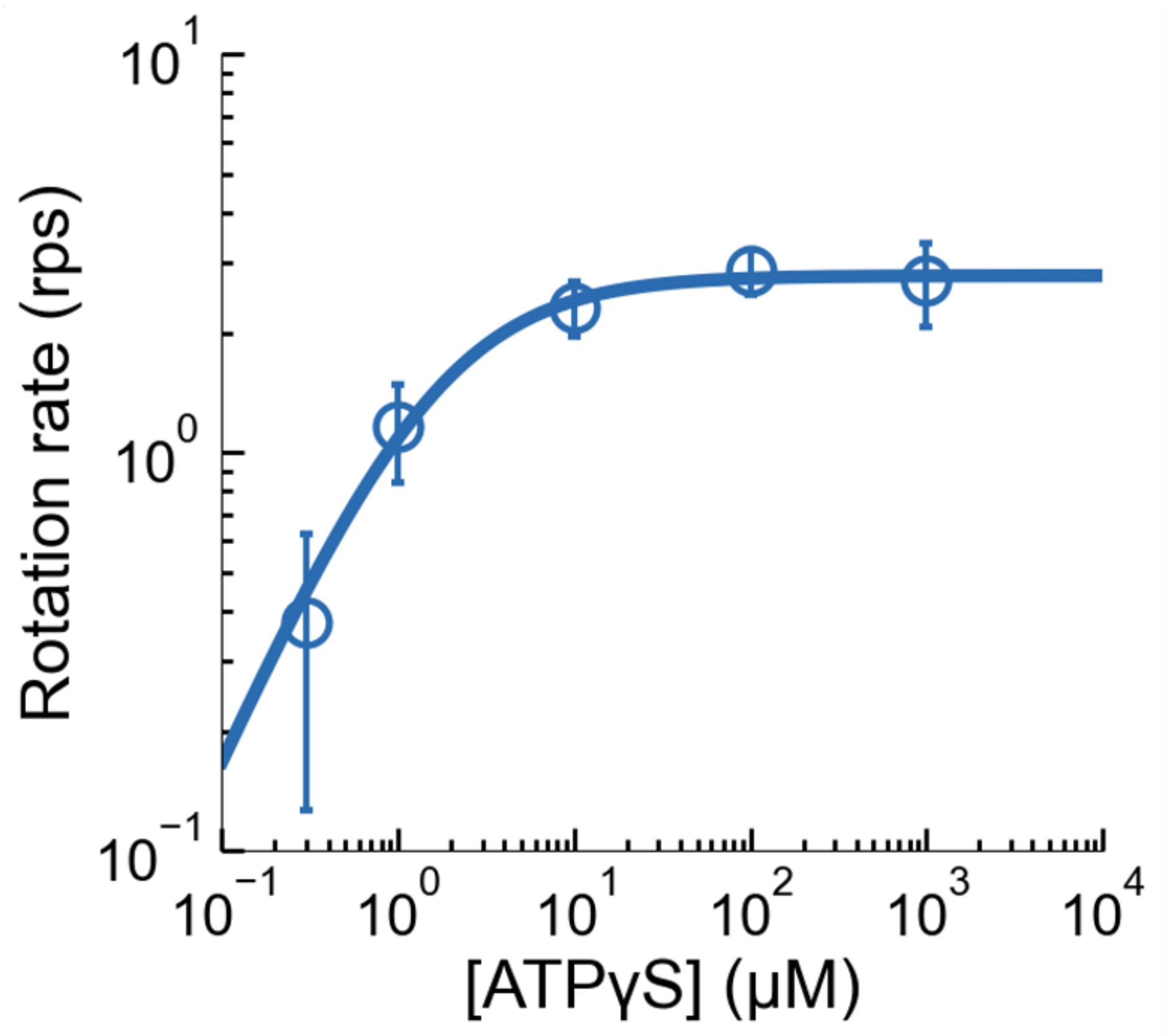

Fig. S5. [ATPγS] versus rotation rate.

[ATPγS] is plotted against the rotation rate for axle-less TF_1. The mean and SD of each data point are shown as circles and error bars, respectively (n = 4). Solid lines indicate Michaelis–Menten fits. V_{max}: 2.8 ± 0.1 rps, K_m: 1.6 ± 0.2 μM. Values are shown as fitted parameter ± fitting error. The ATPγS-binding rate constant (k_{on}), estimated as 3 × V_{max}/K_m, was 5.3×10^7 M^{-1} s^{-1}.

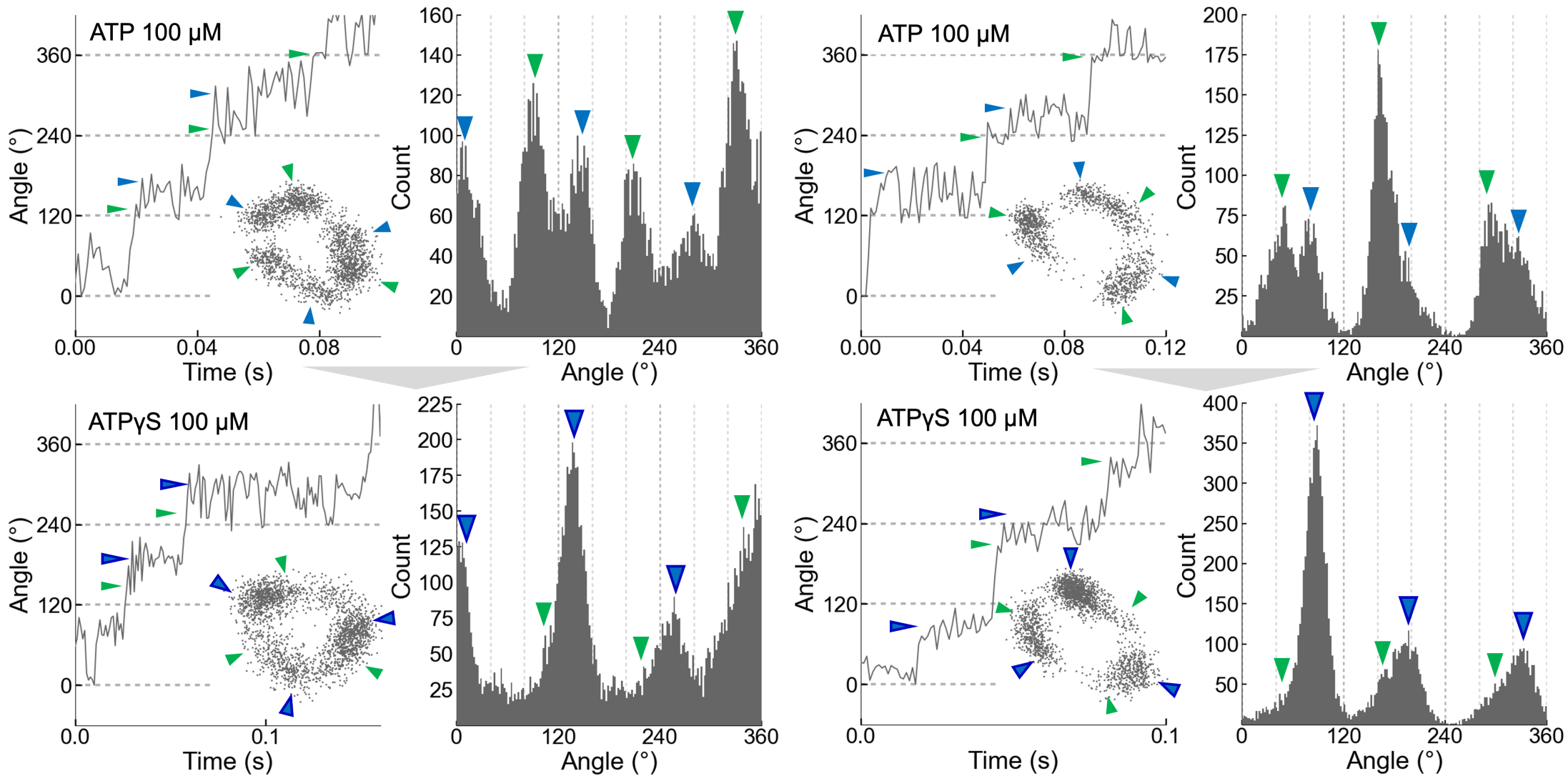

Fig. S6. Identification of the catalytic dwell from buffer exchange experiments, related to Fig. 4.

Rotation time courses and angular histograms for the same molecule during a buffer exchange experiment, in which the substrate was switched between ATP (top) and ATPγS (bottom) at concentrations above K_m. The x–y plots are shown as insets. Two molecules are shown as examples. Pauses indicated by blue arrowheads were identified as catalytic dwells. Pauses indicated by green arrowheads were termed the “new dwell,” an additional dwell observed in axle-less TF_1. Videos were recorded at 1,000 fps.

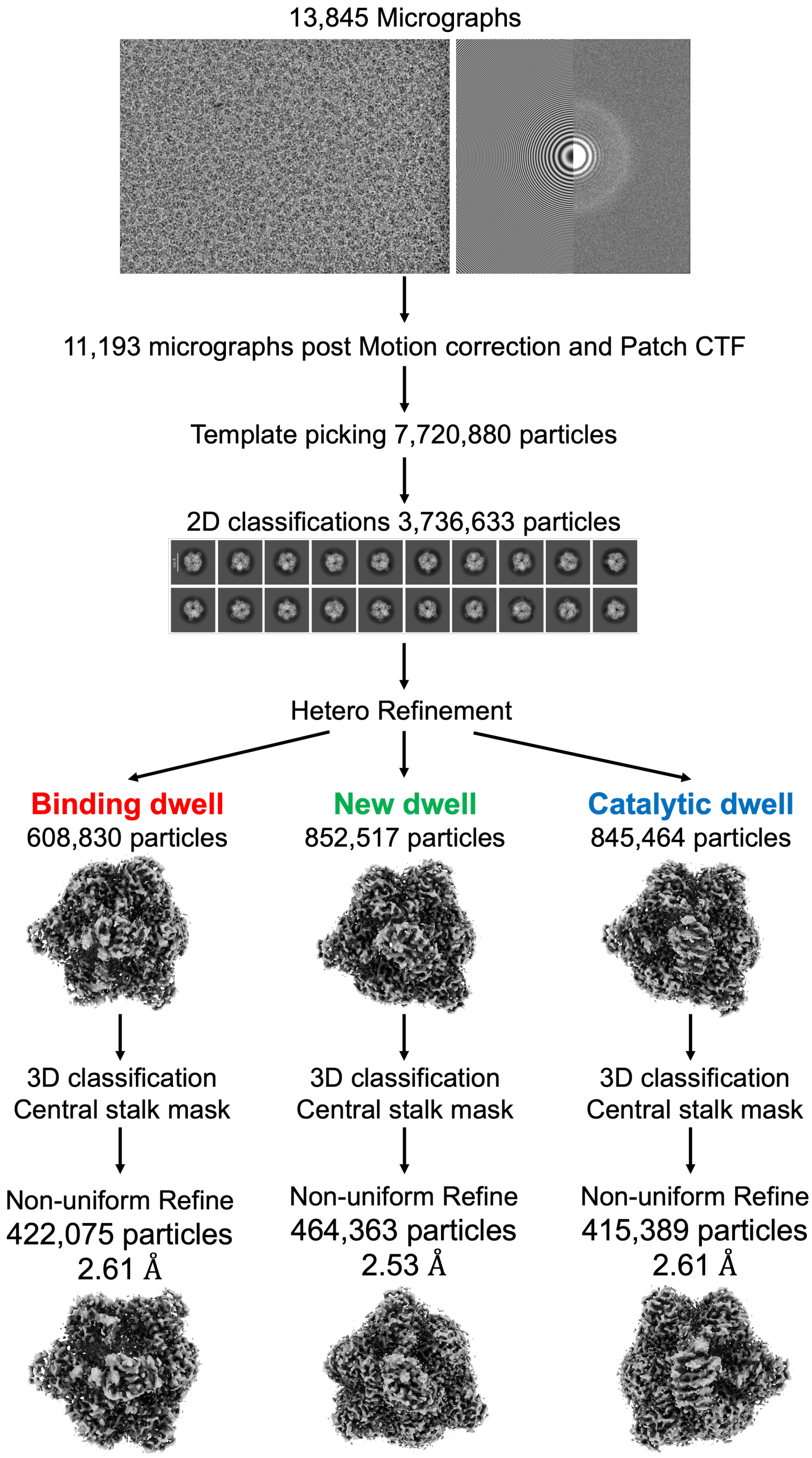

Fig. S7. Cryo-EM data collection and data processing flowchart.

The white scale bar in the 2D class-average images corresponds to 100 Å. The dataset was separated into substates using heterogeneous refinement.

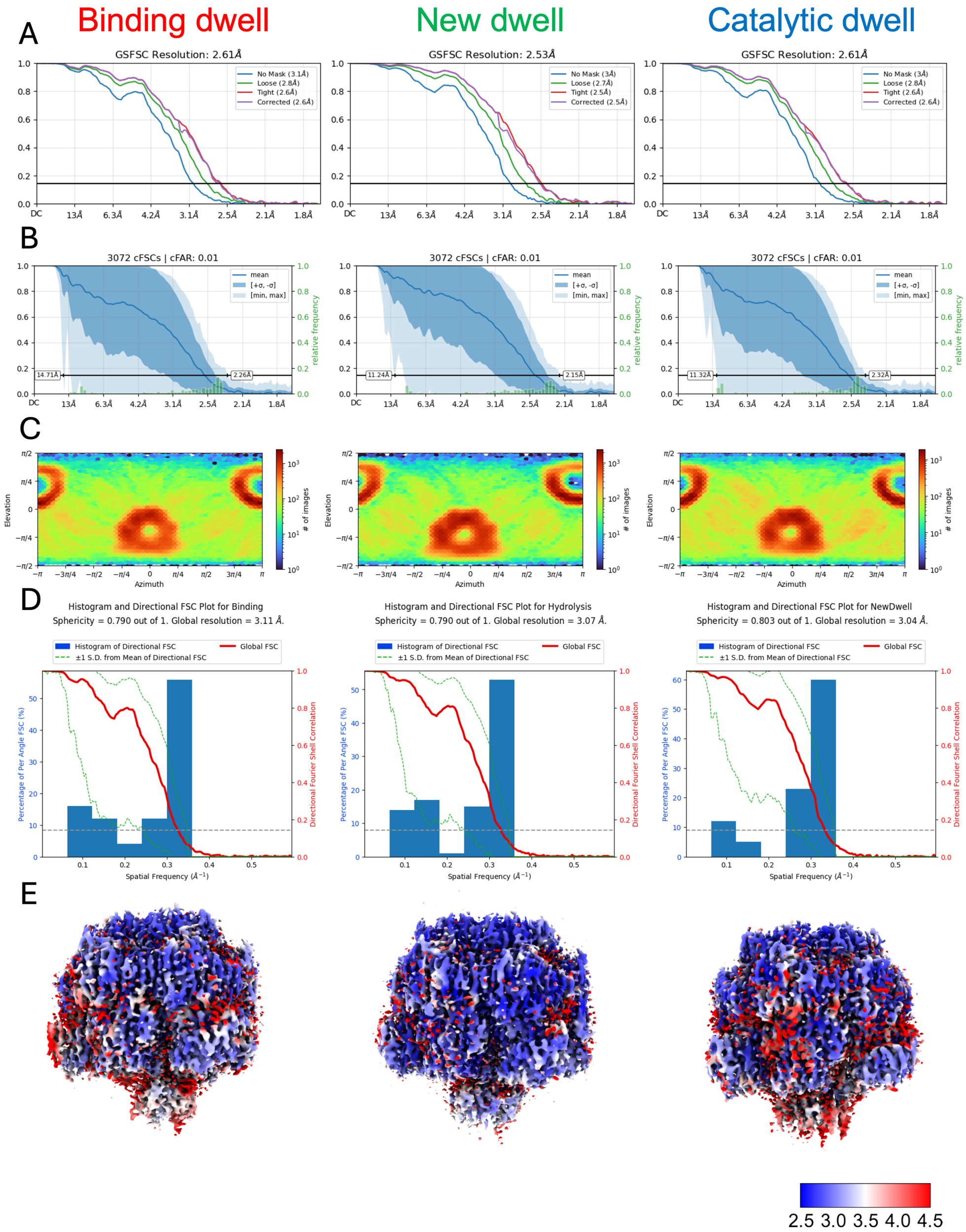

Fig. S8. 3D FSC Curves and Local Resolution Estimates.

(A) Gold-standard Fourier shell correlation (GSFSC) curves calculated in cryoSPARC. (B) Conditional Fourier shell correlation (cFSCs) curves calculated in cryoSPARC. (C) Viewing direction distribution plot. (D) Histograms and direct FSC plots from 3D FSC

analysis. (E) Local-resolution estimates calculated in cryoSPARC. The lower-right panel shows the local-resolution scale.

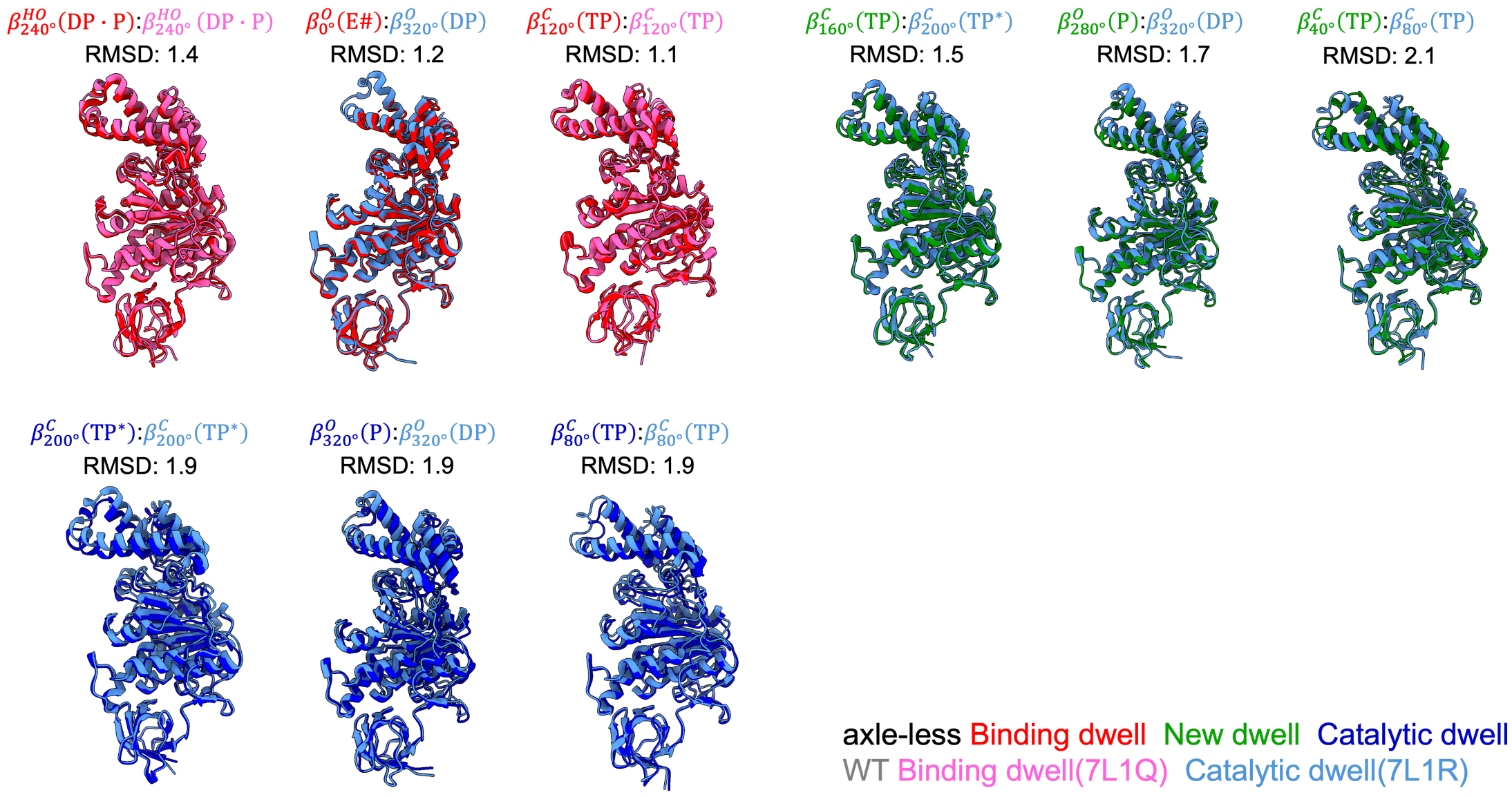

Fig. S9. Comparison of β conformations in axle-less TF_1.

The β subunit structures of axle-less TF_1 were aligned with the corresponding β structures of TF_1, with a focus on the N-terminal ~81 residues that form the β-barrel. The axle-less TF_1 binding dwell, new dwell, and catalytic dwell structures are shown in red, green, and blue, respectively, whereas the TF_1 binding dwell (PDB ID: 7L1Q) and catalytic dwell (PDB ID: 7L1R) structures are shown in pink and light blue, respectively. Note that $\beta_{0°}$ in the axle-less TF_1 binding dwell structure is closer to $\beta^{O}_{320°}$ in 7L1R than to the corresponding $\beta^{HC}_{0°}$ in 7L1Q. This feature was also observed in the ATP-waiting state of TF_oF_1[2]. This is likely because the binding dwell structure obtained here reflects the ATP-waiting state itself rather than the binding dwell structure that reflects the temperature-sensitive (TS) reaction.

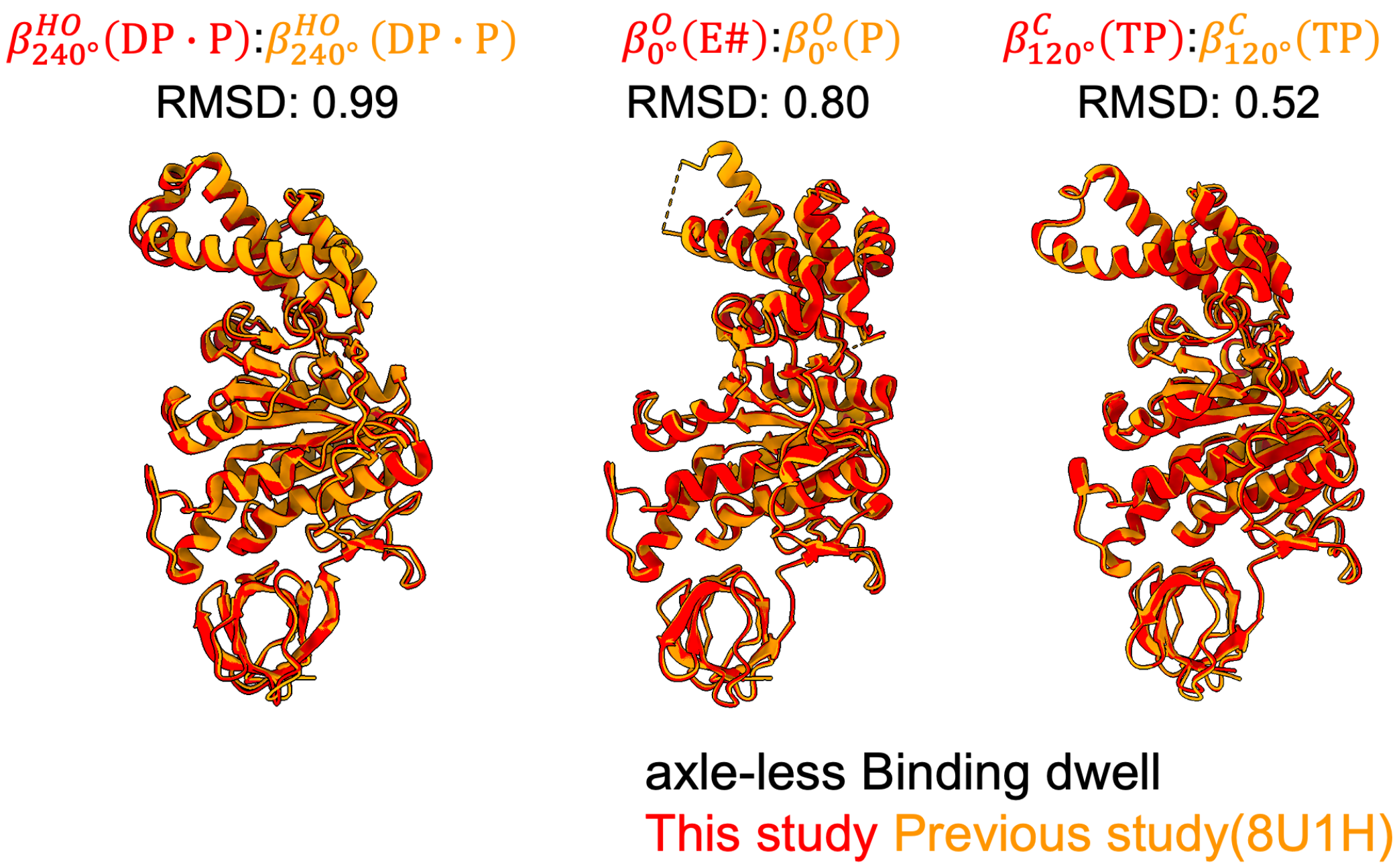

Fig. S10. Comparison of binding dwell β conformations between the axle-less TF_1 structure determined in this study and the previous study.

The binding-dwell β-subunit structures of axle-less TF_1 determined in this study were aligned with the corresponding β structures of previously reported axle-less TF_1, with a focus on the N-terminal ~81 residues that form the β-barrel. The structures determined in this study and in the previous study are shown in red and orange, respectively. The two structures were in good agreement.

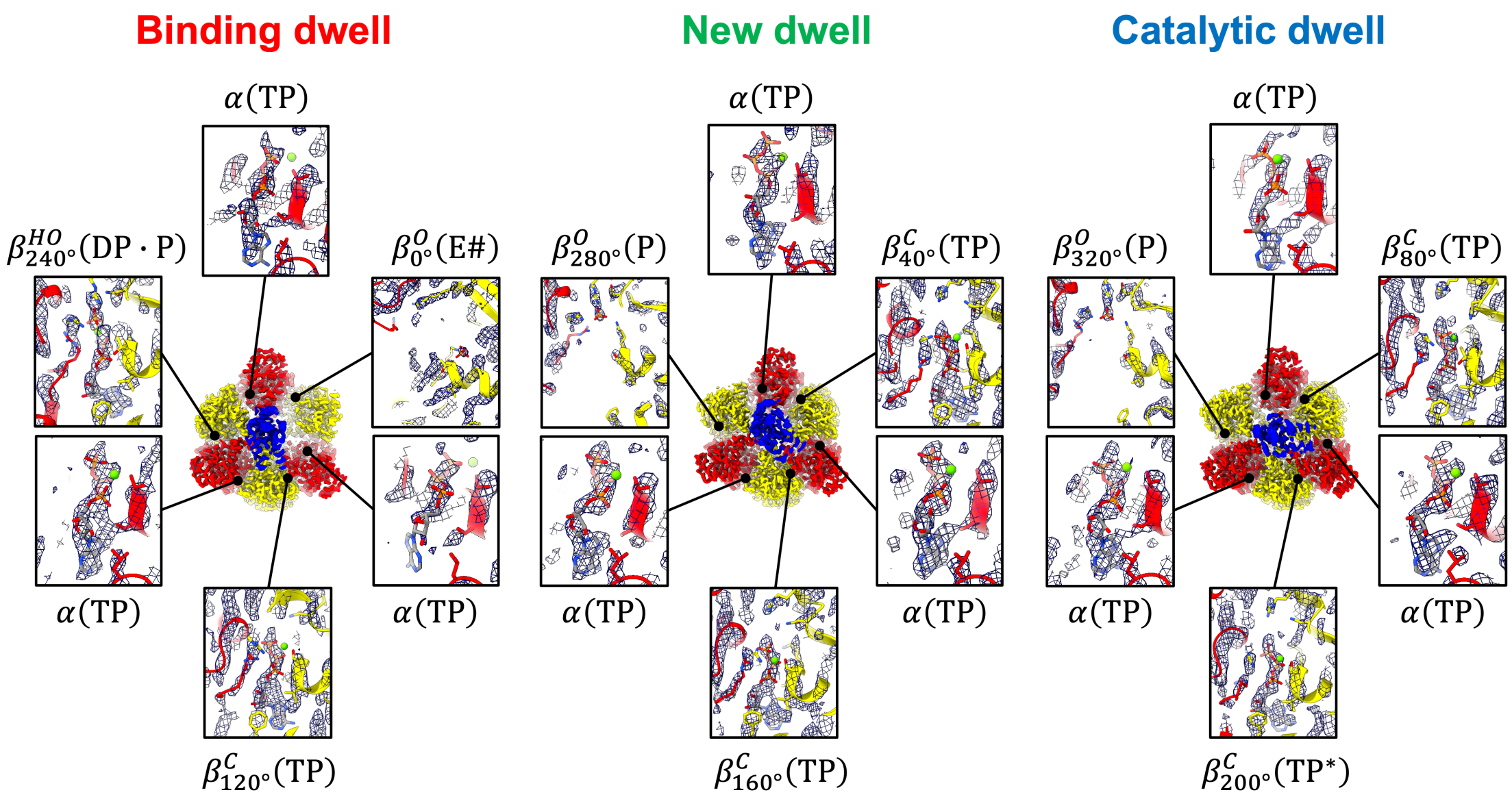

Fig. S11. Close-up views of the nucleotide binding sites.

Cryo-EM maps are shown together with close-up views of each nucleotide binding site. All α subunits in all dwells bound nucleotides.

In the binding dwell, the canonical nucleotide-binding site of $\beta_{0°}$ was empty, whereas weak additional density was observed at a noncanonical phosphate-binding position and was tentatively modeled as Pi. This state is therefore denoted E#. The equivalent site in WT TF_1 was ATP-bound[3]. This difference is likely because the binding-dwell structure captured here reflects an ATP-waiting state rather than a binding-dwell state that reflects the TS reaction. In the catalytic dwell, $\beta_{320°}$ was nucleotide-free, whereas the equivalent site in TF_1 was ADP-bound[3]. This difference is likely because the ADP

rebinding inferred in TF_1 did not occur in axle-less TF_1. The nucleotide state in the new dwell closely matched that in the catalytic dwell.

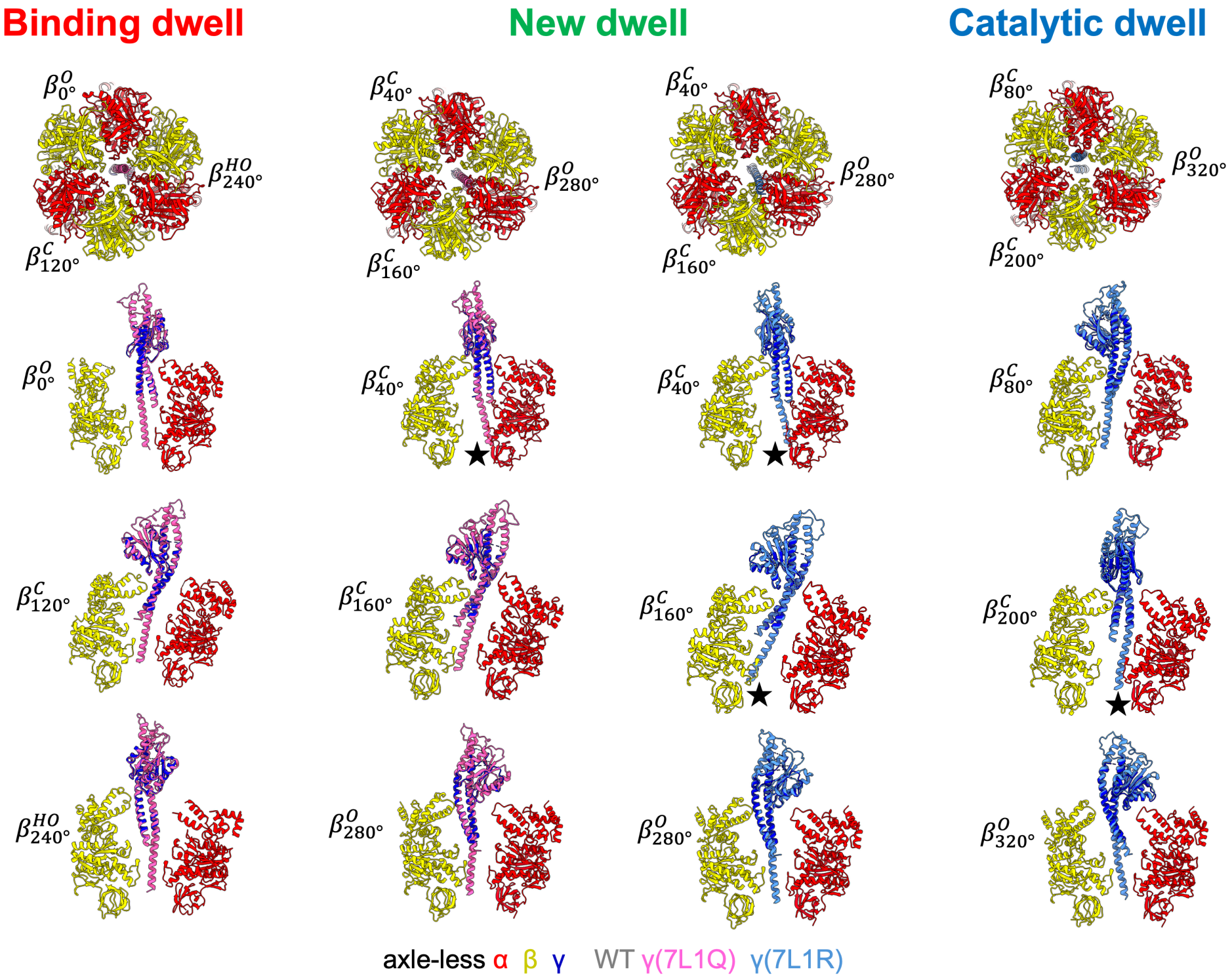

Fig. S12. Steric clashes between γ and αβ upon alignment of TF_1 γ onto axle-less TF_1 γ.

The TF_1 γ subunit structures were aligned with the corresponding γ structures of axle-less TF_1, focusing on the N-terminal residues 12–45 and the C-terminal residues 221–257 that form the axial helix. In the representation, the α, β, and γ subunits of axle-less TF_1 are shown in red, yellow, and blue, respectively. The γ subunit of TF_1 in the binding dwell (PDB ID: 7L1Q) is shown in pink, and the γ subunit of TF_1 in the catalytic dwell (PDB ID: 7L1R) is shown in light blue. In the axle-less TF_1 binding dwell, the aligned TF_1 γ did not show steric clashes with α and β. In the catalytic dwell, the aligned TF_1 γ clashed with the α subunit facing $\beta_{200°}$. In the new dwell, the aligned TF_1 γ from the TF_1 binding dwell clashed with the α subunit facing $\beta_{40°}$, and the aligned TF_1 γ from

the TF_1 catalytic dwell clashed with the α subunit facing β_{40° and with β_{160°. Sites of steric clash are indicated by black stars.

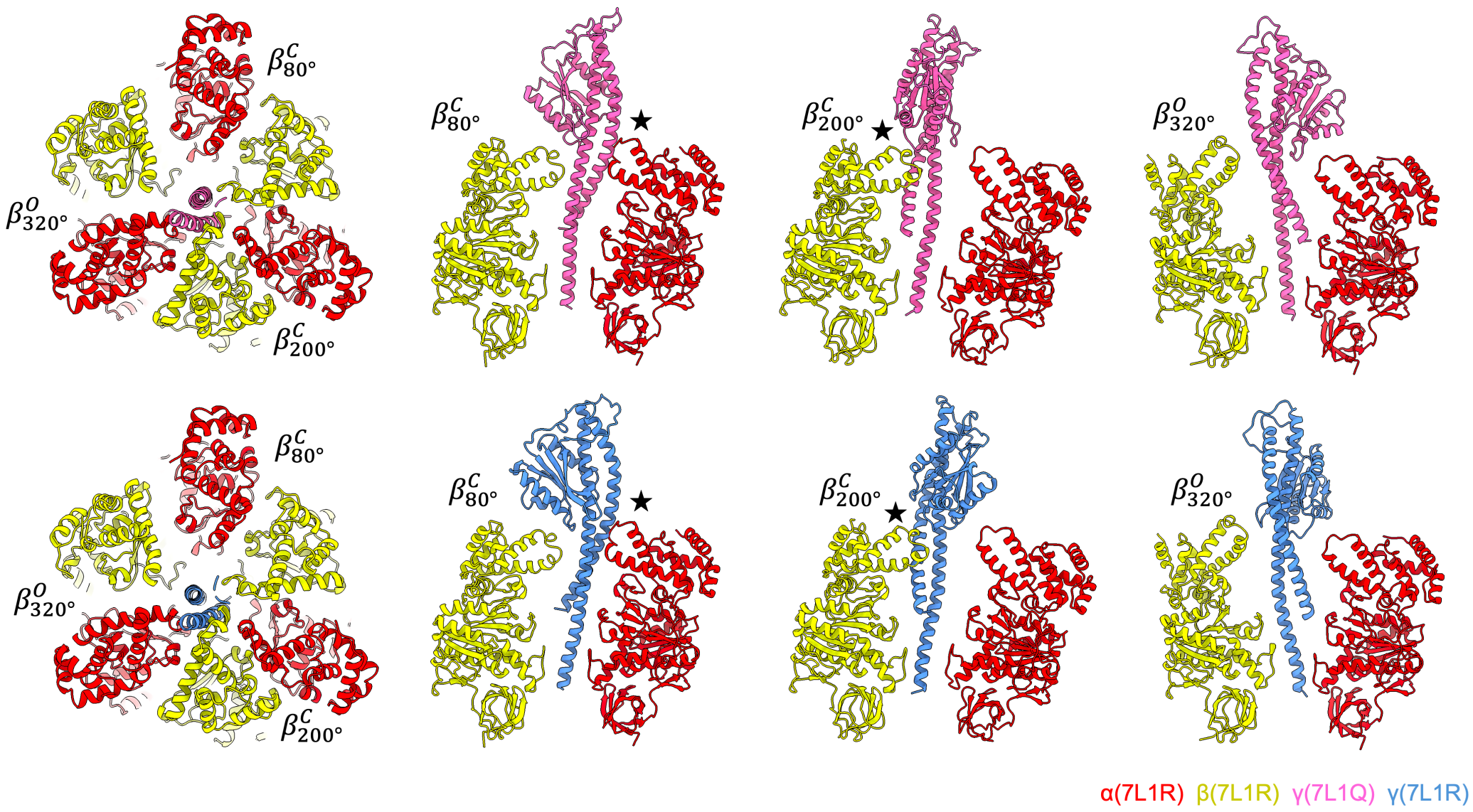

Fig. S13. Steric clash between the TF_1 catalytic dwell $\alpha_3\beta_3$ ring and γ subunits at 40° rotational angle.

The TF_1 γ subunit was rotated relative to the $\alpha_3\beta_3$ ring by +40° for the binding dwell structure and by −40° for the catalytic dwell structure. In the representation, the α and β of TF_1 in the catalytic dwell (PDB ID: 7L1R) are shown in red and yellow, respectively. The γ subunit of TF_1 in the binding dwell (PDB ID: 7L1Q) is shown in pink, and the γ subunit of TF_1 in the catalytic dwell (PDB ID: 7L1R) is shown in light blue. Both γ clashed with the α subunit facing $\beta_{80°}$ and with $\beta_{200°}$. Sites of steric clash are indicated by black stars.

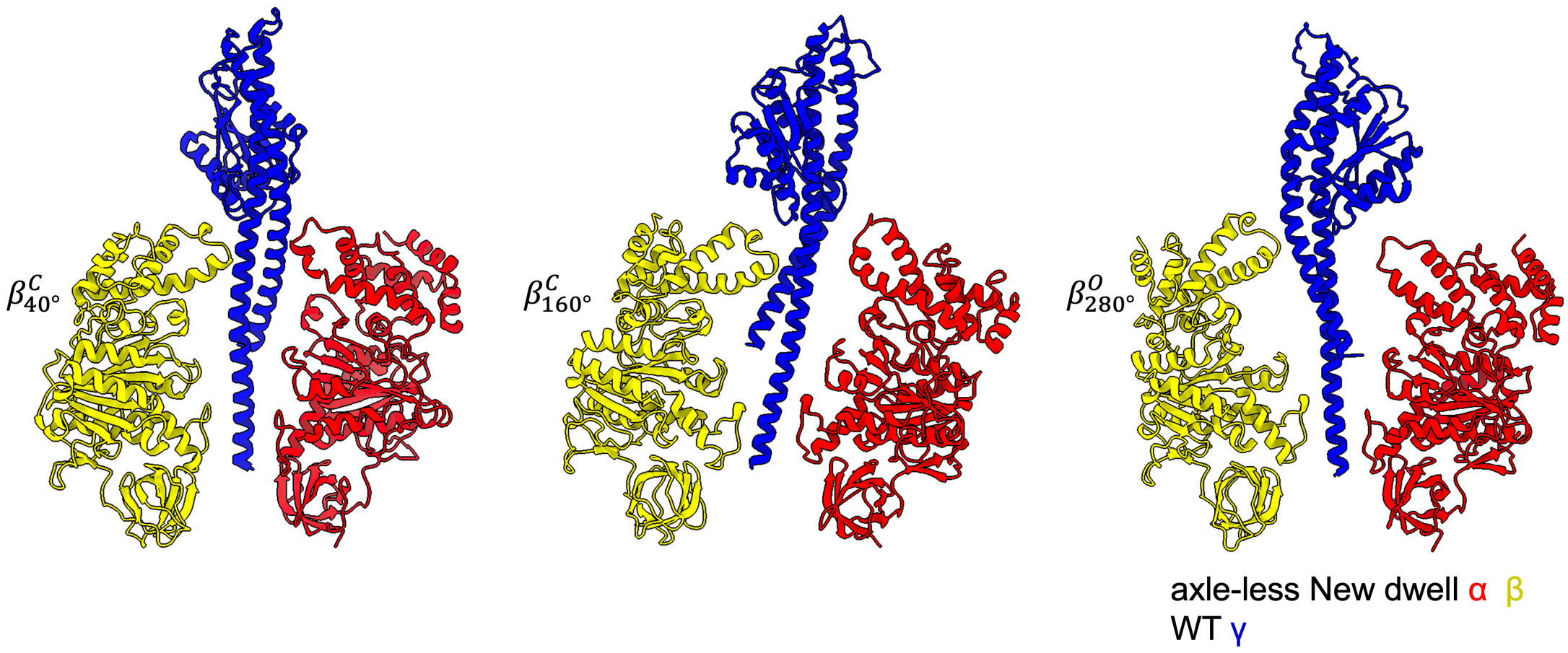

Fig. S14. Fitting of full-length γ in the new-dwell $\alpha_3\beta_3$ conformation of axle-less TF_1.

WT TF_1 γ was fitted onto the $\alpha_3\beta_3$ ring of axle-less TF_1 in the new dwell. The γ structure represents an intermediate generated by linear interpolation between the binding dwell structure (PDB ID: 7L1Q) and the catalytic dwell structure (PDB ID: 7L1R). In the representation, the α, β, and γ subunits are shown in red, yellow, and blue, respectively.

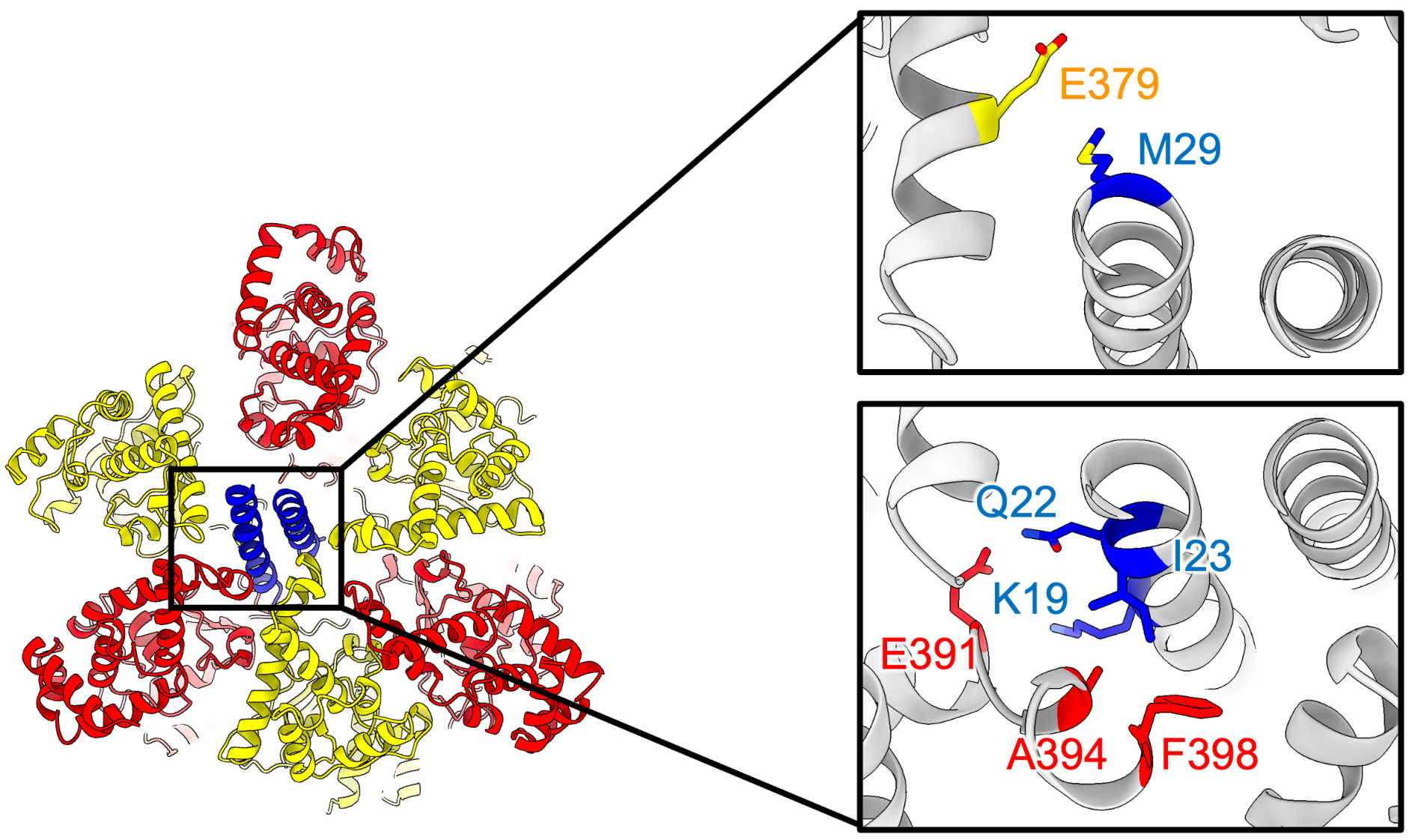

Fig. S15. New interactions between α/β and γ in the new dwell.

The α and β residues detected within 4.5 Å of γ in the new dwell, together with the corresponding γ residues, are shown. The upper-right panel shows the β–γ interactions, and the lower-right panel shows the α–γ interactions. The α, β, and γ subunits are colored red, yellow, and blue, respectively.

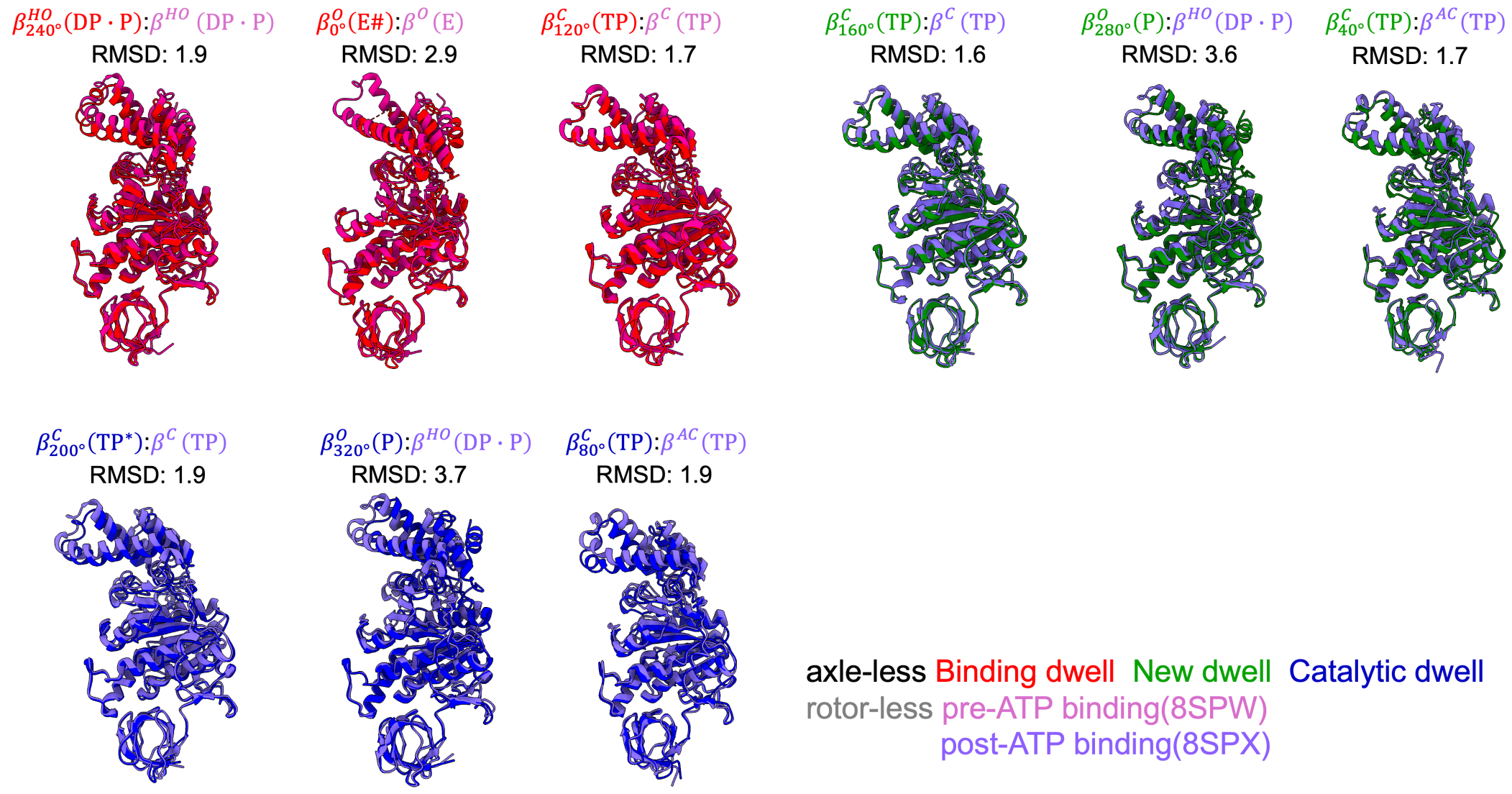

Fig. S16. Comparison of β conformations in axle-less TF_1 and rotorless TF_1.

The β subunit structures of axle-less TF_1 were aligned with the corresponding β structures of rotorless TF_1, with a focus on the N-terminal ~81 residues that form the β-barrel. The axle-less TF_1 binding dwell, new dwell, and catalytic dwell structures are shown in red, green, and blue, respectively, whereas the rotorless TF_1 pre-ATP binding (PDB ID: 8SPW) and post-ATP binding (PDB ID: 8SPX) structures are shown in red-purple and blue-purple, respectively. The binding-dwell structure of axle-less TF_1 was compared with the pre-ATP-binding structure of rotorless TF_1, whereas the new-dwell and catalytic-dwell structures of axle-less TF_1 were compared with the post-ATP-binding structure of rotorless TF_1. The β subunit structures of axle-less TF_1 and rotorless TF_1 were well aligned overall. However, the β subunits corresponding to $\beta_{280°}/\beta_{320°}$

adopted an open conformation in axle-less TF_1, whereas they remained in a half-open conformation in rotorless TF_1.

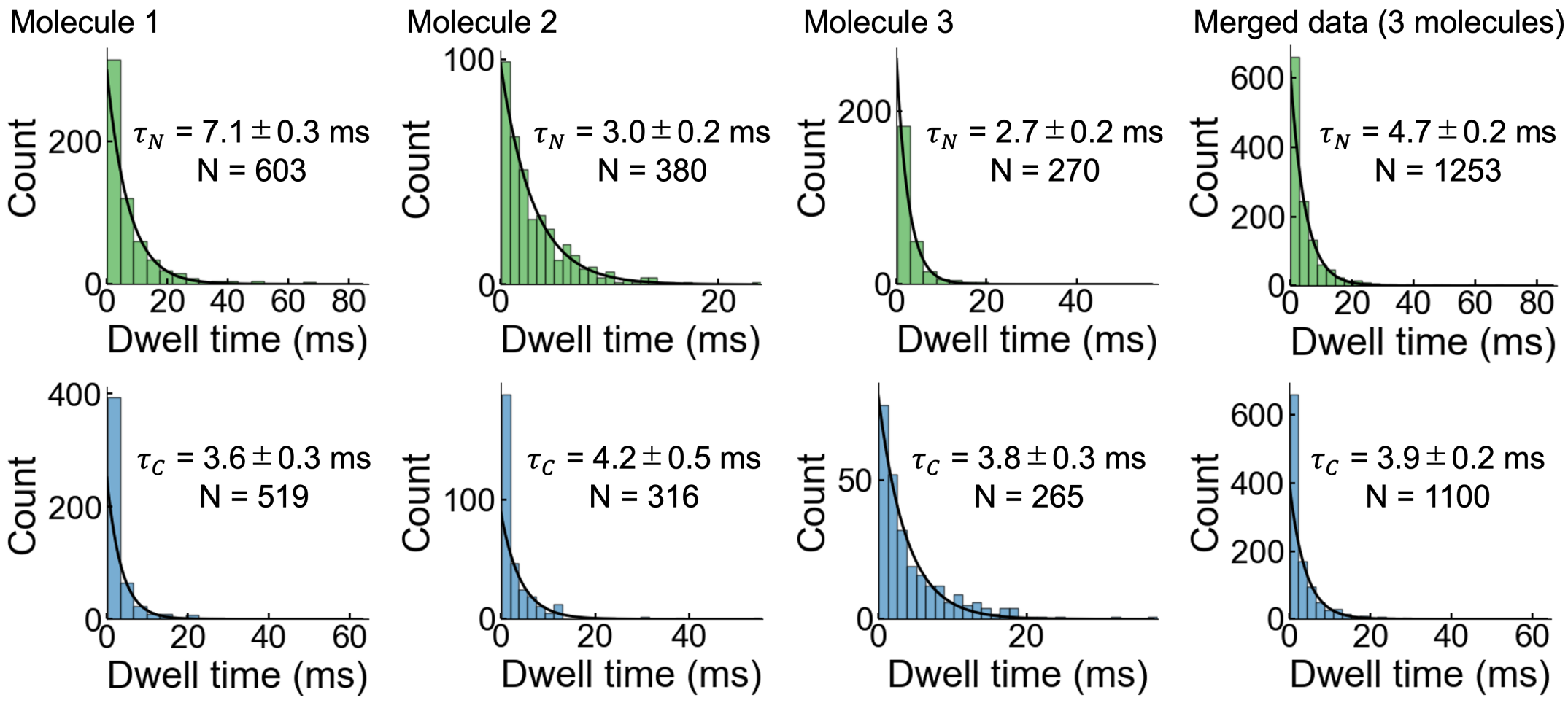

Fig. S17. Molecule-by-molecule dwell-time analysis of the new and catalytic dwells.

Dwell-time histograms for the new dwell (top, green) and catalytic dwell (bottom, blue) are shown for each of the three analyzed molecules and for the merged data. Black curves indicate single-exponential fits. The fitted time constants τ_N and τ_C and the number of dwell events (N) are indicated in each panel. The fitted τ_N values varied among molecules from 2.7 to 7.1 ms, whereas the τ_C values ranged from 3.6 to 4.2 ms. The ΔG values calculated independently for the three molecules ranged from -0.65 to $0.52 k_B T$, with all values falling within $\pm 0.7 k_B T$ of zero (Table S2). Values are shown as fitted parameter ± fitting error. Videos were recorded at 30,000 fps in the presence of 100 μM ATP.

Table S1. Cryo-EM data collection, refinement and validation statistics.

	#1 Binding Dwell (EMD-69782)	#2 New Dwell (EMD-69783)	#3 Catalytic Dwell (EMD-69784)
**Data collection and processing**			
Magnification	59,000x	59,000x	59,000x
Voltage (kV)	300	300	300
Electron exposure (e–/Å^2)	74	74	74
Defocus range (μm)	0.5-1.5	0.5-1.5	0.5-1.5
Pixel size (Å)	0.84	0.84	0.84
Symmetry imposed	C1	C1	C1
Initial particle images (no.)	608,830	852,517	845,464
Final particle images (no.)	422,075	464,363	415,389
Map resolution (Å) masked FSC threshold 0.143	2.61	2.53	2.61
Map resolution (Å) unmasked FSC threshold 0.143			
Global resolution (Å)	3.11	3.04	3.07
Sphericity (threshold 0.5)	0.790	0.803	0.790
Map sharpening *B* factor (Å^2)	-61.1	-60.6	-62.0

Table S2. Parameters used to estimate the free-energy bias between the new and catalytic dwells for individual molecules and merged data.

Molecule	τ_N (ms)	τ_C (ms)	n_f	n_b	P_f	ΔG $(k_B T)$
Mol 1	7.1	3.6	63	366	0.15	0.52
Mol 2	3.0	4.2	64	175	0.27	–0.65
Mol 3	2.7	3.8	27	176	0.13	–0.48
Merged	4.7	3.9	154	717	0.18	–0.01

References

1. Watanabe RR, Kiper BT, Zarco-Zavala M, Hara M, Kobayashi R, Ueno H, García-Trejo JJ, Li CB, Noji H (2023) Rotary properties of hybrid F1-ATPases consisting of subunits from different species. iScience 26.

2. Nakano A, Kishikawa J, Mitsuoka K, Yokoyama K (2023) Mechanism of ATP hydrolysis dependent rotation of bacterial ATP synthase. Nat. Commun. 14:4090.

3. Sobti M, Ueno H, Noji H, Stewart AG (2021) The six steps of the complete F1-ATPase rotary catalytic cycle. Nat. Commun. 12.
